# Drug repositioning targeting glutaminase reveals drug candidates for the treatment of Alzheimer’s disease patients

**DOI:** 10.1186/s12967-023-04192-6

**Published:** 2023-05-20

**Authors:** Abdulahad Bayraktar, Xiangyu Li, Woonghee Kim, Cheng Zhang, Hasan Turkez, Saeed Shoaie, Adil Mardinoglu

**Affiliations:** 1 Centre for Host-Microbiome Interactions, Faculty of Dentistry, Oral & Craniofacial Sciences, King’s College London, London, SE1 9RT UK; 2 Bash Biotech Inc, 600 West Broadway, Suite 700, San Diego, CA 92101 USA; 3 Science for Life Laboratory, KTH–Royal Institute of Technology, SE-17121 Stockholm, Sweden; 4 Department of Medical Biology, Faculty of Medicine, Atatürk University, Erzurum, Turkey

**Keywords:** Alzheimer’s disease, Profile-based computational drug repositioning, Glutaminase, Anti-carcinogenic drugs, Gene co-expression network analysis, Parbendazole

## Abstract

**Background:**

Despite numerous clinical trials and decades of endeavour, there is still no effective cure for Alzheimer's disease. Computational drug repositioning approaches may be employed for the development of new treatment strategies for Alzheimer’s patients since an extensive amount of omics data has been generated during pre-clinical and clinical studies. However, targeting the most critical pathophysiological mechanisms and determining drugs with proper pharmacodynamics and good efficacy are equally crucial in drug repurposing and often imbalanced in Alzheimer’s studies.

**Methods:**

Here, we investigated central co-expressed genes upregulated in Alzheimer’s disease to determine a proper therapeutic target. We backed our reasoning by checking the target gene’s estimated non-essentiality for survival in multiple human tissues. We screened transcriptome profiles of various human cell lines perturbed by drug induction (for 6798 compounds) and gene knockout using data available in the Connectivity Map database. Then, we applied a profile-based drug repositioning approach to discover drugs targeting the target gene based on the correlations between these transcriptome profiles. We evaluated the bioavailability, functional enrichment profiles and drug-protein interactions of these repurposed agents and evidenced their cellular viability and efficacy in glial cell culture by experimental assays and Western blotting. Finally, we evaluated their pharmacokinetics to anticipate to which degree their efficacy can be improved.

**Results:**

We identified glutaminase as a promising drug target. Glutaminase overexpression may fuel the glutamate excitotoxicity in neurons, leading to mitochondrial dysfunction and other neurodegeneration hallmark processes. The computational drug repurposing revealed eight drugs: mitoxantrone, bortezomib, parbendazole, crizotinib, withaferin-a, SA-25547 and two unstudied compounds. We demonstrated that the proposed drugs could effectively suppress glutaminase and reduce glutamate production in the diseased brain through multiple neurodegeneration-associated mechanisms, including cytoskeleton and proteostasis. We also estimated the human blood–brain barrier permeability of parbendazole and SA-25547 using the SwissADME tool.

**Conclusions:**

This study method effectively identified an Alzheimer’s disease marker and compounds targeting the marker and interconnected biological processes by use of multiple computational approaches. Our results highlight the importance of synaptic glutamate signalling in Alzheimer’s disease progression. We suggest repurposable drugs (like parbendazole) with well-evidenced activities that we linked to glutamate synthesis hereby and novel molecules (SA-25547) with estimated mechanisms for the treatment of Alzheimer’s patients.

**Supplementary Information:**

The online version contains supplementary material available at 10.1186/s12967-023-04192-6.

## Introduction

Alzheimer’s disease (AD) is becoming one of the most prevalent public health threats due to incrementally ageing populations. A wide range of novel compounds have been proposed to treat AD in numerous clinical trials in the past decade, but most of them failed or showed limited success. Thus, exploring the new use of the previously approved and investigational molecules by using a computational drug repurposing (CDR) approach together with a systems medicine approach may provide a promising solution. In general, the pharmacokinetics, bioavailability and possible side effects of repurposed drugs have been well-characterised, which simplifies the regulatory procedures for drugs and reduces the cost and duration compared to the de novo drug development [1]. For instance, a series of drugs that have been previously approved for the treatment of hypertension, diabetes, epilepsy, nausea and several other diseases have undergone clinical trials for the treatment of AD [2–5]. These drugs remarkably attenuated AD symptoms in early phases by improving numerous clinical parameters other than directly targeting tau or amyloid-beta. This endorses the conceptual shift in the amyloid-centred definition of AD and the transition to drug repositioning in the field [6].

CDR usually benefits an integrated analysis of large-scale biological, biomedical, and electronic health-related data by the use of high computation capabilities and state-of-the-art algorithms. The richness of data resources has resulted in various CDR strategies. Among them, the profile-based CDR strategy supposes that pharmacologic perturbations caused by small molecules are comparable even under varying biological conditions since repurposed small molecules share similar therapeutic mechanisms or modes of action (MOAs) [1]. Furthermore, no prior knowledge of drugs or diseases is required, making the profile-based approach widely applicable. The massive increase in gene expression profiles and resources allows for an increase in profile-based drug repositioning studies [7–11].

The Connectivity Map (CMap) is an extensive example of a resource for the high-throughput transcriptomics profiles [9]. The database includes gene expression signature profiles of different human cell lines perturbed by thousands of compounds, gene overexpression or inhibition. The mapping of drug-perturbed perturbation and disease-induced perturbation on human cells is a commonly used profile-based method to discover potentially effective drugs against diseases. In this method, a drug is considered to be useful if it can potentially reverse the disease-induced gene expression dysregulation [1, 12]. This approach has been successful in drug discovery studies in clear cell renal carcinoma, liver cancer, SARS-COV-2, and AD [13–16]. However, a drug repurposed in this way usually has multiple gene targets mixed by disease driver and passenger genes, limiting the identification and validation of mechanisms of drug effect.

In this study, to identify the therapeutic gene targets, we referred to our preliminary systems biology study, where genetic and metabolic changes occurring in the AD brain were examined using gene co-expression network (GCN) analysis and genome-scale metabolic modelling [17]. Then, we applied our previously proposed drug repositioning method which aims to repurpose useful drugs for targeting a specific gene based on the similarity analysis of the perturbations induced by the target gene knockdown and candidate drug treatment on the available human cell lines [13, 18]. To our knowledge, drug repositioning studies using perturbagens are targeted to a limited set of drugs on certain cell lines, where the condition occurs; this brings two handicaps: (i) drug repositioning is under-performing due to being restricted on under-studied cell lines, (ii) fewer compounds are screened and restrains the potential solutions for many conditions including the AD [19–21]. The use of multiple cell lines is a good practice preferred in cancer cell lines thanks to the similar drug MOAs on multiple diseases [22]. Hence, we applied the drug repositioning approach based on the transcriptomics data from multiple non-glial/non-neural tissues. Further computational tests have been performed to investigate the target gene druggability, candidate drug bioavailability, functional enrichment of drug-perturbed profiles and target gene-drug associations. Finally, we conducted in vitro experiments to test and validate the drug's efficacy and toxicity.

## Materials and methods

All computational analyses were performed on Rstudio version 4.1 for macOS.

### Identification of AD druggable target genes

Hub genes (i.e. genes present in multiple modules and having a high degree in all) from co-expression modules that have been revealed for the dorsolateral prefrontal cortex, temporal cortex and cerebellum were identified using preliminary study findings [17]. Significantly up-regulated genes were filtered referring to data from the same study (log-fold change > 0 and False Discovery Rate < 0.05. number of genes passing criteria for dorsolateral prefrontal cortex: 1472; temporal cortex: 205; cerebellum: 944).

Gene knockout and drug-induced perturbagens (or signature profiles) were downloaded from the Connectivity Map database (see Availability of data and materials). The matrices were split into cell lines to speed up computations. To determine targetable genes, available knockdown perturbagens for filtered genes were screened from the metadata of signature profiles (cellinfo_beta.txt: 240 cell lines in total, 158 of them from 20 known lineages; siginfo_beta.txt: 177518 shRNA/gene knockout profiles and 572269 drug profiles).

The essentialities of the drug target gene and other possibly targetable genes were evaluated using DepMap data (DepMap 22Q1 Public + Score, Chronos) [23] that was filtered for 117 cell lines from the central nervous system and peripheral nervous system lineages and 371 cell lines from seven tissues (skin, lung, kidney, liver, colon, breast, prostate) whose target gene knockdown perturbagens exists. The essentiality scores of a target gene and other possibly targetable genes measured for the nervous system and the essentiality scores of the target gene measured for seven tissues were plotted.

### Drug identification by using a drug repositioning approach

**Fig. 1:**
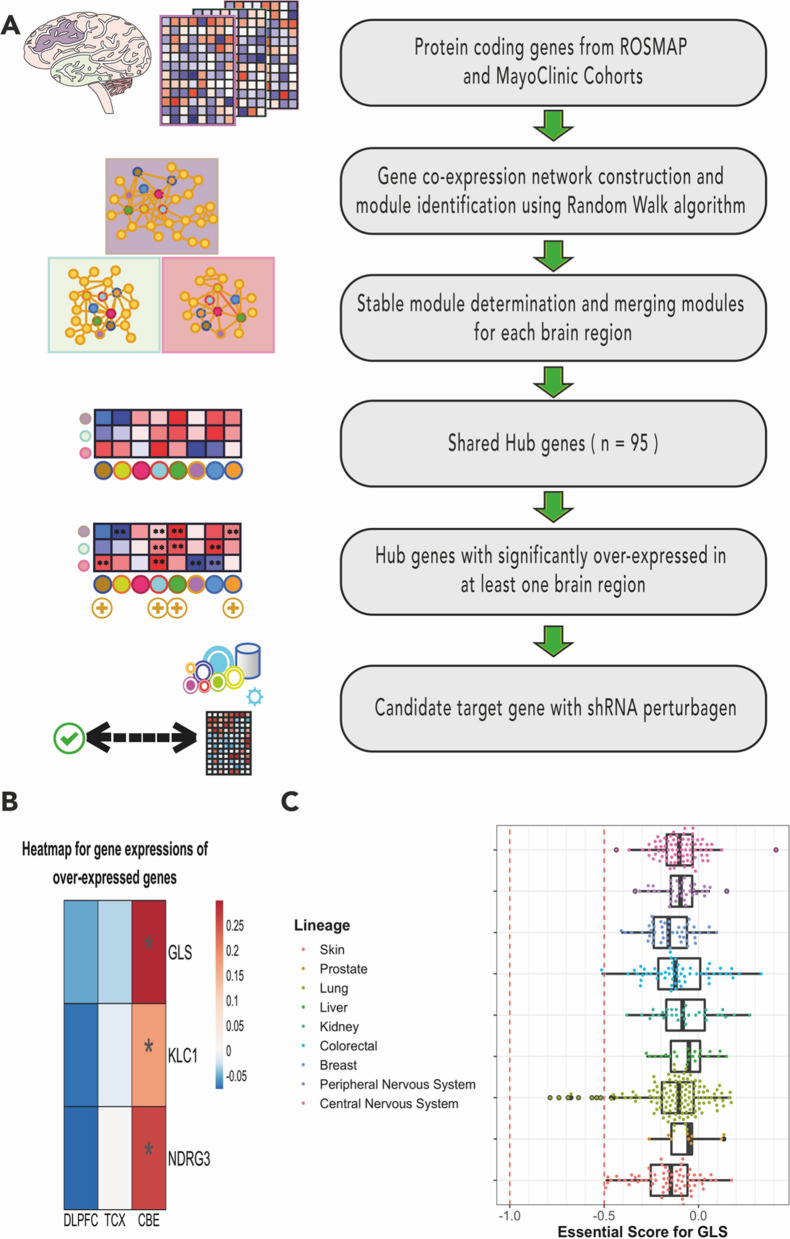
**A** The flowchart illustrating the identification of candidate target genes. To summarise, transcriptome data from Religious Orders Study/Memory and Ageing Project (ROSMAP) and MayoClinic cohorts sampled from the dorsolateral prefrontal cortex (purple in the illustration), temporal cortex (light green in the illustration) and cerebellum (light red in the illustration) of the AD patients and non-AD elders have been analysed by systems biology methods, including GCN. 95 shared and central co-expressed genes were in our interest. Hub genes overexpressed at least in one brain region in AD were used for further analysis. **B** Heatmap showing the log-twofold changes of *GLS*, *KLC1* and *NDRG1*, which have been overexpressed either on the disease dorsolateral prefrontal cortex, temporal cortex or cerebellum. The blue-to-red colour transition indicates overexpression in AD samples. Significant differential expressions (adjusted p-value < 0.05) labelled with “*”. **C** Boxplot showing the *GLS* essentiality scores in nervous system cell lineages. Boxes represent the interquartile ranges where a median is a middle vertical line in a box

shRNA gene knockdown and drug-induced perturbagens were attained from the CMap database as GCTX files. They were split into smaller files for each cell line to speed up the calculations (see Availability of data and materials). Each perturbagen was composed of 12328 genes identically. shRNA-induced perturbagens for the target gene and drug-induced perturbagens from the same cell lines were extracted using the cmapR package [24]. Target gene knockout perturbagens were screened from all available cell lines, found in seven: skin (A375), lung (A549), kidney (HA1E), liver (HEPG2), colon (HT29), breast (MCF7) and prostate (PC3). In total, 24539 drug-induced perturbagens corresponding to these cell lines were screened. Correlation matrices were constructed by calculating the Spearman correlation coefficients between combinations of drug-perturbed and target-specific shRNA-perturbed perturbagens for each cell line, subsequently filtered for the optimal dose and time point with the highest correlation coefficients. Drugs with the highest correlations in the top 1% were identified, ranked for each cell line, and termed as top 1% drugs. The first set of drugs was determined as the top 1% of drugs from at least six cell lines (n = 4). The second set of drugs was determined as the top 1% of drugs from at least two cell lines with the best median rank (n = 4). The drug determination workflow was illustrated in Fig. 1.

Next, the bioavailability of candidate drugs, i.e. absorption, distribution, metabolism and excretion, were investigated using the SwissADME web tool [25]. On this web tool, we evaluated gastrointestinal bioavailability, the blood–brain barrier (BBB) permeability, P-glycoprotein-provided resistance, and drug degradation via inhibiting Cytochrome P450 subunits (CYPs). PAINS [26], Brenk [27], leadlikeness [28] and synthetic accessibility features given in the output were evaluated for future insights into these compounds (Table 3).

### Knockdown and drug profile pathway enrichment

Pathway enrichment was analysed for the shRNA-induced profiles of drug target gene (*GLS*) and drug-induced profiles of candidate drugs (n = 8), a subset of best-correlated drugs (n = 100) and randomly selected drugs (n = 100) with respect to MSigDB hallmark pathways (n = 50) and KEGG pathways (n = 347). Pathway gene sets were accessed using the EnrichmentBrowser package [29]. EntrezIDs and gene symbol conversions were done using the AnnotationDBI package [30]. GSEA was performed using the fgsea package [31] with 10000 permutations. Profile pathway enrichments for each profile from different cell lines were combined using metapro package [32]; Benjamini–Hochberg adjusted p-values (adj. p-values) were combined by the weighted Fisher’s method whereas normalised enrichment scores (NES) were combined by the weighted Z-method. Due to the higher power of the weighted Fisher’s method on unassociated p-values (from unassociated experiments), NES with adj. p-value higher than 0.05 were converted to zero, reflecting their low strength. Adjusted p-values (padj) were converted to Reliability as:

$$Reliability = - \log \left( {padj} \right)$$

Pathway enrichments were visualised as bubble plots.

### Network analysis of drug targeting mechanisms

CMap data for drug annotations, Cheng’s PPI dataset and the Human Reference Interactome PPI dataset were downloaded [33, 34]. Using CMap data, drugs and target proteins were tabulated as the DPI dataset. We integrated the DPI dataset and two PPI datasets which have been created again from multiple sources meticulously, to build an interactome (12057 unique DPIs and 262549 PPIs, connecting 4383 compounds and 16996 proteins; Table 1 & Additional file 1: Fig S5) allowing for the depiction of deep drug-protein associations.

**Table 1:** Table displaying features of resource datasets integrated into the interactome

Interaction type	Link	Entity count in network	Entity count in the database
Drug-protein interaction	Connectivity Map	4383 compounds	6798 compounds
		2183 protein or target gene	2183 proteins
		1124 modes of action	1437 modes of action
		12057 interactions	12058 interactions
Protein–protein interaction	HuRI—The Human Reference Interactome	8229 proteins	9094 proteins
		52129 interactions	64006 interactions
	Cheng F et al	15934 proteins	16677 proteins
		217019 interactions	243603 interactions

Interactome was built using the igraph package [35]. We generated subnetworks from the interactome separately for each MOA associated with candidate drugs by setting the maximum length of paths as four (drug <  =  > drug target <  =  > neighbour of target gene <  =  > target gene) (i) to emphasise the most direct drug-target associations and (ii) to reduce computational expenses. These subnetworks were imported to Cytoscape using the RCy3 package for better examination of target protein-drug associations and visualisation [36].

### In vitro validation of candidate drug pharmacodynamics

The toxicity, induced protein level changes and efficacy of two candidate drugs (parbendazole and bortezomib) and a disease reference drug (memantine) were examined on the glioblastoma cell line U138MG.

#### Cell culture

Before performing these assays, we decided on the optimum cell count per well, by comparing the optical density of absorbance for L-glutamate (ab120049) between DMEM-0819 (with glutamine) and DMEM-5671 (without glutamine) culture media, which was tested by glutamate assay kit from Abcam (Abcam PLC, Cambridge, UK) at 450 nm following its protocol (Additional file 1: Fig S6). Accordingly, we cultured 20000 cells per well in DMEM-0819 with L-alanyl-L glutamine, adherently, treated with drugs dissolved in DMSO separately. To prepare LDH assay high control samples, cell lysis solution was added instead of drugs. Samples were incubated in a humidified incubator (at 37 ℃, 5% CO2). A microplate reader was used to measure absorbances.

#### Western blot

Drug-induced glutaminase content was measured by Western blotting. The cells were washed with PBS and lysed with CelLytic M (C2978, Sigma-Aldrich, Saint Louis, MO, USA) lysis buffer containing protease inhibitors. The lysates were centrifuged at 12,000 rotations per minute for 5 min, and the supernatant was collected. Proteins were separated by Mini-PROTEAN^®^ TGXTM Precast Gels (BioRad, Berkeley, CA, USA) and transferred to a Trans-Blot Turbo Mini 0.2 um PVDF Transfer Packs membrane (BioRad, Berkeley, CA, USA) by using Trans-Blot^®^ TurboTM Transfer System (Bio-Rad, Berkeley, CA, USA). The antibodies for mitochondrial glutaminase-1 (kidney-type) and glutaminase-2 (liver-type) encoded from two GLS isomers and GAPDH were used for primary immunoblotting. All the antibodies were diluted at a 1:1000 concentration. DMSO was used as a blank reference. The membranes were incubated in primary antibody solution overnight at 4 ℃ with gentle rocking. The secondary antibody, goat Anti-Rabbit HRP (ab205718) or goat anti-mouse IgG-HRP (sc2005, Santa Cruz Biotechnology, Inc., Dallas, TX, USA), was blotted for 30 min at 4 ℃ with gentle rocking. The protein bands were detected and band densities were quantified by Image J software.

#### Drug toxicity

Tetrazolium reduction assay (MTT assays) and lactate dehydrogenase release assays (LDH assays) were conducted to assess cell metabolic activity (cell proliferation) and to measure cytotoxicity (drug toxicity). Abcam protocols (MTT: ab211091, LDH: ab65293) were followed. Here, we summarised both protocols.

Before adding assay solutions on day 1 (24 h) and day 2 (48 h), the drug treatment media were aspirated from plates and transferred to well plates.

For the MTT assay, serum-free media and MTT Reagent were added to each well. Control samples were prepared without using cells but serum-free media and MTT reagent. Well plates were incubated at 37 °C for 3 h, MTT Solvent was added to each well, and the wells were wrapped in foil and shaken on an orbital shaker for 15 min. The reduction of MTT by mitochondrial succinate dehydrogenase was measured by optical density (OD) absorbance at 590 nm (yellow to purple). Cytotoxicity (CT) was calculated as a ratio of measures of absorbance of control wells (AbsC) and absorbance of sample-containing wells (AbsS) as below:

$$CT = 100*\frac{{\left( { AbsC - AbsS } \right)}}{AbsC}$$

For the LDH release assay, the LDH reaction mix was added to wells. Low control samples were prepared only using cells. Well plates were incubated at 37 °C for 30 min. The release of LDH from cells due to damaged plasma membrane was measured by reading absorbance at OD = 450 nm (blue). Cytotoxicity (CT) was calculated as a ratio of measures of OD absorbance of low control wells (AbsLC), high control wells (AbsHC) sample wells (AbsS) as below:

$$CT = 100* \frac{{\left( { AbsS - AbsLC } \right)}}{{\left( { AbsHC - AbsLC} \right)}}$$

#### Glutamate contents assay

Drug efficacy describes how the drug-protein interaction manipulates the normal action of the protein. Glutamate content was examined at differing drug concentrations with the glutamate assay kit to measure drug efficacies. Abcam protocol (ab83389) was followed to quantitate glutamate content. Here, we summarised the protocols.

To prepare samples, cells were harvested, washed with cold PBS, resuspended in an assay buffer, homogenised, incubated for 15–30 min on ice, and centrifuged for 2–5 min at 4 °C at top speed using a cold microcentrifuge. The supernatant was collected and transferred to a clean tube, then distributed to well plates.

To quantitate free glutamate levels, the reaction mixture was added to each standard and sample well. The background reaction mix was added to the background sample wells. Wells were mixed and incubated at 37 °C for 30 min protected from light. The colour change was measured at OD absorbance at 450 nm (blue). Glutamate concentration was calculated as formulated in the protocol, then divided by relative cytotoxicity by MTT assay to extrapolate drug efficacy.

## Results

### ***Systems biology methods highlight the centrality of glutaminase over-expression in AD***

**Fig. 2:**
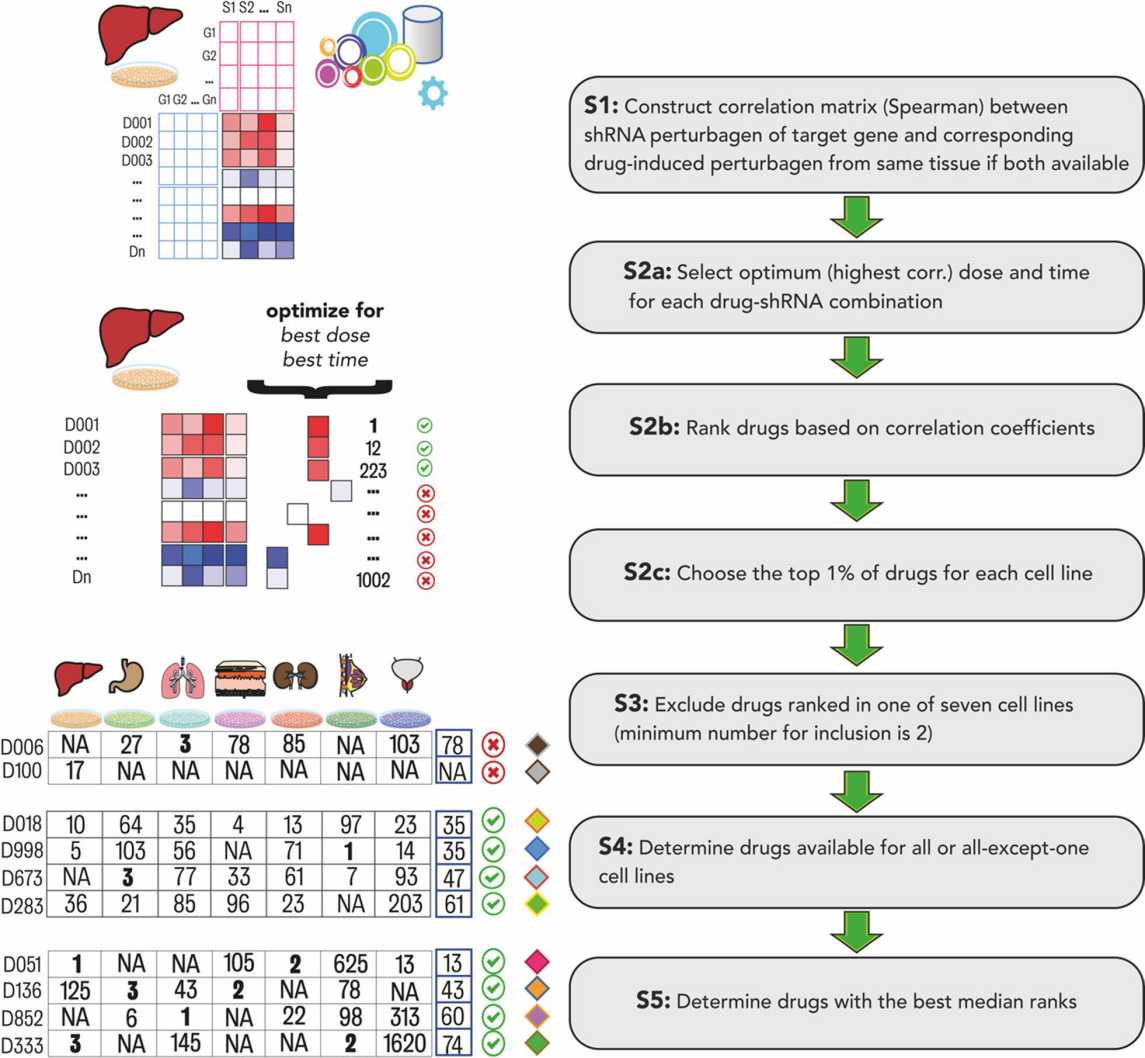
The workflow of drug repositioning methodology

As shown in Fig. 2A, human brain transcriptome profiles from the dorsolateral prefrontal cortex, temporal cortex and cerebellum have been analysed by the use of GCN analysis in our previous study, showing co-expression patterns of potential pathology-driving genes, which is consistent through the diseased brain [17]. As a result, we found that 95 genes were shared by the functional modules derived from multiple brain regions. Furthermore, we focused on up-regulated genes that could be suppressed by drugs, since drug-induced overexpression is more likely to increase drug resistance which is specifically risky for elderly people [37]. Among these 95 genes, only *GLS, KLC1* and *NDRG1* were significantly up-regulated in the AD brain (Fig. 2B) and only *GLS* had available gene knockdown profiles in the CMap database, which could be used for the drug repositioning analysis.

We checked the essentiality of *GLS* in the nervous system to ensure its targetability. Cancer Dependency Map (DepMap) stores extensive genomic information about cancer and regular cell lines screened for CRISPR gene knockdowns. Powerful algorithms are used in the database to deduce gene knockdown fitness effects to estimate the gene-dependent survivability [23, 38]. An essential score closer to − 0.5 represents knockdown-dependent depletion, indicating higher essentiality for cell proliferation and a score closer to − 1 represents knockdown-dependent obliteration, implying high essentiality and a positive score may indicate cell proliferation. In cancer research, it is promising to target the genes with low essential scores, whereas a neurodegeneration study should target genes non-essential for cell proliferation with high essential scores hypothetically [39]. Our screening of 117 cell lines of nervous system lineage showed that *GLS* had a high essential score as observed in [39], confirming glutaminase targetability in the brain. Supporting this, *GLS* knockdown was not predicted to inhibit cell proliferation necessarily in other tissues (n = 371) (Fig. 2C). A comparison of essential scores for other co-expressed genes in the nervous system highlights the necessity of this check (Additional file 1: Fig S1).

*GLS* encodes glutaminase (i.e., glutaminase-1 or kidney-type glutaminase) that is located in the mitochondrial intermembrane and hydrolyzes glutamine to glutamate and ammonium which is the primary reaction for glutamate biosynthesis [40]. Glutamate can be further converted to other amino acids (arginine, aspartate, isoleucine, leucine, serine, valine, tyrosine), α-ketoglutarate, which is a member of the citric acid cycle, and glutathione, which is a universal antioxidant [41]. More importantly, glutamate is the most abundant excitatory neurotransmitter, stimulating post-synaptic N-methyl-D-aspartate receptors (NMDARs). Hence, glutamate is vital for protein metabolism, energy production and transmitting information across the central nervous system. Normal glutaminase activity is essential for brain health.

However, excess glutamate release from presynaptic regions and glutamate spillover can activate extrasynaptic NMDARs and presynaptic metabotropic glutamate receptors, disrupting ion balance via Ca^++^ influx, leading to regression of dendritic spines and reductions in synaptic glutamate transmission, ultimately ending in neuron loss. Glutaminase hyperactivity is neurotoxic due to the increase of glutamate and decrease of glutamine concentrations [42, 43]. Supporting this, we have reported high levels of glutamine and glutamate in the AD brain fuelling the glutamate excitotoxicity based on the reporter metabolite analysis [17]. All these pieces of evidence emphasise the danger of glutaminase overexpression in Alzheimer’s and the hypothesis that glutaminase suppression and glutaminase-dependent reduction in glutamate may be therapeutic for AD.

### Profile-based drug repositioning puts forth anti-cancer drugs and novel compounds against glutaminase

In our proposed drug repositioning method, we hypothesize that a drug has an inhibitory effect on a target gene if the drug treatment led to expressional changes highly positively correlated to expressional changes caused by the knockdown of this target gene [13]. We screened for drugs targeting *GLS* as explained in Methods. Primarily, we considered drugs with the best representation. Mitoxantrone was the only drug represented (correlations at the top 1%) in all seven cell lines, whereas crizotinib, bortezomib and withaferin-a were represented in six cell lines. However, pharmacokinetic models accessible in the SwissADME web tool predicted that these anti-cancer drugs were unlikely to reach the brain tissues through the blood–brain barrier (BBB) and more likely to be effluated and degraded by P-glycoproteins and cytochromes P450 (CYP) in the brain, respectively, which diminish their supposed efficacy [25]. Therefore, we gathered an additional set of drugs using less stringent rules as given before. We narrowed the list down to the four best-ranked drugs (parbendazole, BRD-K5433811, BRD-K03641750 and SA-25547), to continue further analyses together with the former four drugs (mitoxantrone, crizotinib, bortezomib, withaferin-a). All candidate drugs were screened by the SwissADME tool again for their bioavailability. Though parbendazole, BRD-K5433811 and SA-25547 were predicted to permeate the BBB, BRD-K5433811 was predicted to be effluated from the brain (Additional file 1: Fig S3).

### Repurposable drugs may re-stabilize cell cycle through glutaminase-linked pathways as shown by enrichment analysis

Perturbagen correlations were the first indication of direct drug effects on glutaminase. While mitoxantrone, bortezomib, withaferin-a, crizotinib and parbendazole had high-to-medium correlations in all cell lines, three novel drugs had medium correlations. Memantine is an NMDAR antagonist and is an often-used drug in the AD treatment [44]. Memantine-perturbed profiles were not correlated to *GLS* knockdown profiles. This implies that NMDA suppression does not translate into less glutaminase activity (Additional file 1: Fig S2).

We performed gene set enrichment analysis (GSEA) on GLS-knockdown and candidate drug profiles to further elaborate on the effect of gene suppression and drug inductions on biological pathways in terms of biological states and processes. We referred to pathways in the Kyoto Encyclopaedia of Genes and Genomes knowledge base (KEGG) [45] and hallmark gene sets from the Molecular Signatures Database (MSigDB) (Liberzon et al. 2015). Genesets from both databases are well-defined and curated; however, have been generated regarding different visions and delineate different biological spaces.

Initially, perturbagens of 100 randomly selected drugs and 100 best-correlated drugs were used for further hallmark pathways analysis based on the MSigDB (Additional file 1: Fig S4) using the weighted Fisher approach (Yoon et al. 2021). We assumed that (i) reliable changes in biological pathways on a multi-tissue scale as a response to small molecules could be captured and (ii) drugs sharing similar biological effects should also share the same pathway enrichment patterns.

**Fig. 3:**
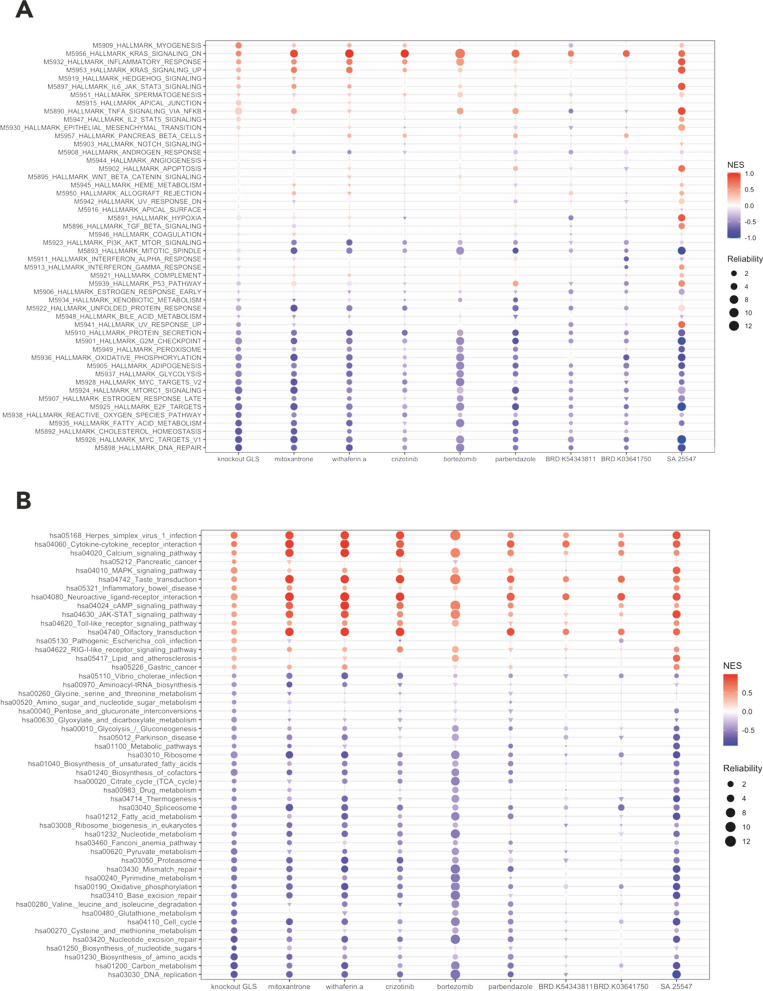
Bubble plots showing the enriched functional pathways regulated by GLS knockdown and candidate drugs, based on (**A**) MSigDB hallmark pathways (n = 50) and (**B**) KEGG pathways significantly changed by GLS knockdown (n = 61). The red colour shows a larger normalised enrichment score (NES)/enrichment, and the blue colour shows a larger negative NES/repression. The size of the “bubble” represents the reliability of enrichment scores, which is calculated as -log(FDR adjusted and weighted Fisher aggregated p-value). The circle represents significant enrichments/repressions, and the triangle represents non-significant enrichment/repressions

It was revealed that energetic metabolism (e.g., oxidative phosphorylation and glycolysis), biosynthetic metabolism (e.g., fatty acid metabolism, adipogenesis, and protein secretion) and cell cycle events (e.g., G2M checkpoint, DNA repair and p53 pathway) were suppressed whereas inflammatory response and various signalling paths (RAS, JAK/STAT and TNFA) were enriched coherently as a response to highly correlated drug inductions (Fig. 3A). These results suggested that these drugs identified by our approach showed a clear suppression effect on the expression of *GLS* and also for candidate drug inductions with note-worthy differences. For instance, mitotic spindle (gene set) repression and Ras signalling pathway enrichment were more reliable and stronger on drug inductions compared to gene suppression, emphasising the effect of these drugs on the cell cycle.

KEGG pathway enrichments added further annotations supporting these patterns, which were often more radical (e.g. calcium and cAMP signalling pathway enrichments) (Fig. 3B). In contrast, enrichment patterns for randomly given drugs were neither significant, strong nor consistent (Additional file 1: Fig S4).

### Different mechanisms of action alter the ubiquitination and activation of glutaminase

We have shown the functional resemblance of candidate drug inductions and glutaminase suppression on biological processes. Nonetheless, causal (i.e. physical) links between molecules need to be shown as well to assure that similar effects do not occur by chance. Network-based approaches can exhibit the physical interactions between drugs and target molecules directly or indirectly in a robust way [46].

Hence, we incorporated this method to augment our understanding of the role of candidate repurposable drugs. CMap Repurposing App (https://clue.io/repurposing-app) provides a curated table of compound metadata including MOAs that they associate, proteins that they bind and clinical indications that they have been used against, displaying such information about candidate drugs. Here, we listed the correlated drugs targeting the same biological entities as in Table 2, disclosing that they have been in use against diseases ranging from gout and fever to rheumatoid arthritis and myeloma. It appeared that five of these candidate drugs have known MOAs, or in other words, it is evidenced that they target a few biological entities or processes: NFkB signalling pathway, topoisomerase, proteasome and ALK tyrosine kinase.

**Table 2:** Table for target MOAs, target proteins (given as UniProt symbols), effective disease areas and effective diseases of candidate drugs (*) and compounds sharing the same target MOAs according to CMAP

Drug name	Target	Target id	Disease area	Indication
Auranofin	NFkB pathway	IKBKB, PRDX5, TRPA1	Rheumatology	Rheumatoid arthritis
BAY-11–7082	NFkB pathway	RELA	Not available	Not available
Betulinic-acid	Apoptosis stimulant NFkB pathway inhibitor	GPBAR1	Not available	Not available
Bindarit	NFkB pathway	CCL2, CCL7, CCL8	Not available	Not available
Bortezomib *	NFkB pathway Proteasome	PSMA1, PSMA2, PSMA3, PSMA4, PSMA5, PSMA6, PSMA7, PSMA8, PSMB1, PSMB10, PSMB11, PSMB2, PSMB3, PSMB4, PSMB5, PSMB6, PSMB7, PSMB8, PSMB9, PSMD1, PSMD2, RELA	Hematologic malignancy	Multiple myeloma MCL
Carfilzomib	Proteasome	PSMA1, PSMA2, PSMA3, PSMA4, PSMA5, PSMA6, PSMA7, PSMA8, PSMB1, PSMB10, PSMB11, PSMB2, PSMB3, PSMB4, PSMB5, PSMB6, PSMB7, PSMB8, PSMB9	Hematologic malignancy	Multiple myeloma
Ceritinib	ALK tyrosine kinase	ALK, FLT3, IGF1R, INSR, TSSK1B	Oncology	NSCLC
Colchicine	Microtubule organization	GLRA1, GLRA2, TUBA1A, TUBA1B, TUBA1C, TUBA3C, TUBA3D, TUBA3E, TUBA4A, TUBB, TUBB1, TUBB2A, TUBB2B, TUBB3, TUBB4A, TUBB4B, TUBB6, TUBB8	Other	Gout Fever
Crizotinib *	ALK tyrosine kinase	ALK, MET	Oncology	NSCLC
Curcumin	Cyclooxygenase Histone acetyltransferase Lipoxygenase NFkB pathway	APP, CA1, CA12, CA14, CA2, CA4, CA6, CA9, CHRM3, CYP3A4, DNMT3B, EP300, MMP13, MMP9, NOS2, PTGS1, PTGS2, XDH	Not available	Not available
Daunorubicin	RNA synthesis Topoisomerase	TOP2A, TOP2B	Hematologic malignancy	AML ALL
Delanzomib	Proteasome	CMA1, CTSG, CYP3A4, ELANE	Not available	Not available
Docetaxel	Tubulin polymerization	BCL2, MAP2, MAP4, MAPT, NR1I2, TUBA1A, TUBA1B, TUBA1C, TUBA3C, TUBA3D, TUBA3E, TUBA4A, TUBB, TUBB1, TUBB2A, TUBB2B, TUBB3, TUBB4A, TUBB4B, TUBB6, TUBB8	Oncology	Breast cancer NSCLC Prostate cancer GAC HNSCC
Doxorubicin	Topoisomerase	TOP2A	Oncology	ALL AML Wilm’s tumour Breast cancer Ovarian cancer Hodgkin’s lymphoma Bladder cancer Multiple myeloma
Epirubicin	Topoisomerase	CHD1, TOP2A	Oncology	•Breast cancer
Erythromycin	NFkB pathway	MLNR	Infectious disease	Listeria Respiratory tract infections Skin infections Syphilis Amebiasis Pelvic inflammatory disease Chlamydia Diphtheria Erythrasma
Idarubicin	Topoisomerase	TOP2A	Hematologic malignancy	AML
Mepacrine	Cytokine production NFkB pathway TP53	PLA2G1B	Infectious disease	Giardiasis
MG-132	Proteasome	PSMB1	Not available	Not available
Mitoxantrone *	Topoisomerase	TOP2A	Neurology/psychiatry	MS Prostate cancer AML
NVP-TAE684	ALK tyrosine kinase	ALK, INSR	Not available	Not available
Oprozomib	Proteasome		Not available	Not available
Paclitaxel	Tubulin polymerization	BCL2, MAP2, MAP4, MAPT, NR1I2, TLR4, TUBA1A, TUBA1B, TUBA1C, TUBA3C, TUBA3D, TUBA3E, TUBA4A, TUBB, TUBB1, TUBB2A, TUBB2B, TUBB3, TUBB4A, TUBB4B, TUBB6, TUBB8	oncology	Ovarian cancer Breast cancer NSCLC
parbendazole *	Tubulin polymerization	TUBB	Not available	Not available
Parthenolide	NFkB pathway	Not available	Not available	Not available
Ro-106–9920	NFkB pathway	Not available	Not available	Not available
Secnidazole	Acetylcholinesterase Microtubule	Not available	Infectious disease	Protozoan infection
Teniposide	Topoisomerase	TOP2A, TOP2B	Hematologic malignancy	ALL
Topotecan	Topoisomerase	TOP1, TOP1MT	Oncology	SCLC Cervical cancer
Ursolic-acid	ATPase NFkB pathway STAT pathway	HSD11B1, PLA2G1B, PTPN1, PYGM	Not available	Not available
Vinblastine	Microtubule Tubuline polymerization	JUN, TUBA1A, TUBB, TUBD1, TUBE1, TUBG1	Oncology	Hodgkin’s lymphoma THL Mycosis SLL Testicular carcinoma Kaposi sarcoma
Secnidazole	Acetylcholinesterase Microtubule	–	Infectious disease	Protozoan infection
Teniposide	Topoisomerase	TOP2A, TOP2B	Hematologic malignancy	ALL

*AML* acute myeloid leukaemia, *ALL* acute lymphoblastic leukaemia, *GAC* gastric adenocarcinoma, *HNSCC* head and neck squamous cell carcinoma, *MCL* mantle cell lymphoma, *MS* multiple sclerosis, *NSCLC* non-small cell lung cancer, *SCLC* small cell lung cancer, *SLL* small lymphocytic lymphoma, *THL* true histiocytic lymphoma

Drug-target interaction is another piece of information provided within this data. We integrated the drug-target interactions dataset and two extensive protein–protein interaction datasets [33, 34], which have been created again from multiple sources meticulously, to build an interactome (12057 unique drug-protein interactions (DPIs) and 262549 protein–protein interactions (PPIs), connecting 4383 compounds and 16996 proteins; Table 1 & Additional file 1: Fig S5) allowing for the depiction of deep drug-protein associations.

**Fig. 4:**
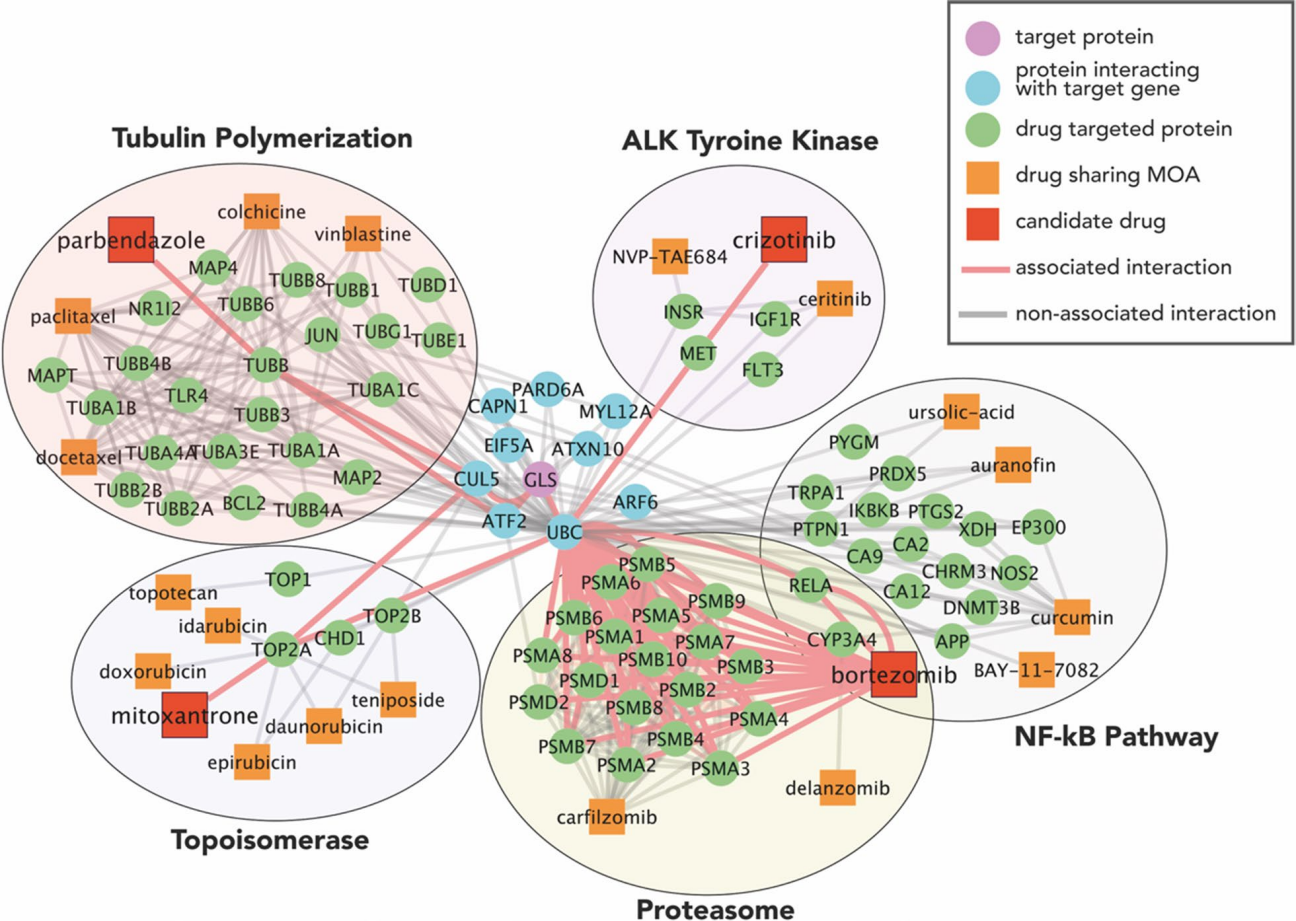
A *GLS*-centred drug-protein interaction network for four candidate repurposable drugs and compounds with a shared mechanism of action. All these drugs indirectly target glutaminase (demonstrated as GLS) as displayed. All drug–target-associated protein connections were examined, and those proteins on the shortest paths (path length = 4) between drugs and target protein are shown. All proteins are represented by their respective protein symbols

After, we zoomed in on the interactome for each MOA associated with candidate drugs by setting the maximum length of paths as four (drug <  =  > drug target <  =  > neighbour of target gene <  =  > target gene) (i) to emphasise the most direct drug-target associations and (ii) to reduce computational expenses. Later, we assembled these subnetworks, revealing that glutaminase has been evidenced to interact physically with several proteins responsible for signal-induced membrane trafficking (ATXN10, ARF6, CAPN1, PARD6, MYL12A), transcriptional activation (transcription factor encoded by ATF2) and ubiquitination of targeted proteins (CUL5, UBC, EIF5A) (Fig. 4). Signal-induced membrane trafficking is complementary to the role of glutamate as the major excitatory neurotransmitter, whereas transcriptional activation and ubiquitination may indicate the regulation of the glutaminase expressions directly. Four candidate drugs interact indirectly with CUL5 or UBC; hence, it can be hypothesised that all four of them may potentially lead to proteolysis of glutaminases.

Proteasomal proteins (PSM family) and NFkB subunit (RELA) interact with UBC and CUL5. From the literature, it is known that the ubiquitin-proteasomal system is a major regulator of protein degradation [47]. The effects of topoisomerases (TOP2A) are less clear. It is known that they can allow gene expressions by unwinding DNA and they may activate the ubiquitin-proteasomal system for target proteins via inducing NF-kB, two opposing mechanisms [48]; however, these proteins are too specific on how they act on their targets that cannot be understood from a non-directed interactome. A similar dichotomy is present for parbendazole-inhibited tubulin (TUBB), which may induce the degradation of glutaminases via CUL5 or activation of their synthesis via ATF2, theoretically.

### ***Repurposable drugs reduce glutamate and glutaminase levels effectively in glial cell cultures***

As a next step, to verify candidate drugs in vitro models, we assessed the toxicity, induced protein levels and efficacy of two selected candidate drugs (parbendazole and bortezomib) and memantine as a positive control on the glioblastoma cell line (U138MG).

**Fig. 5:**
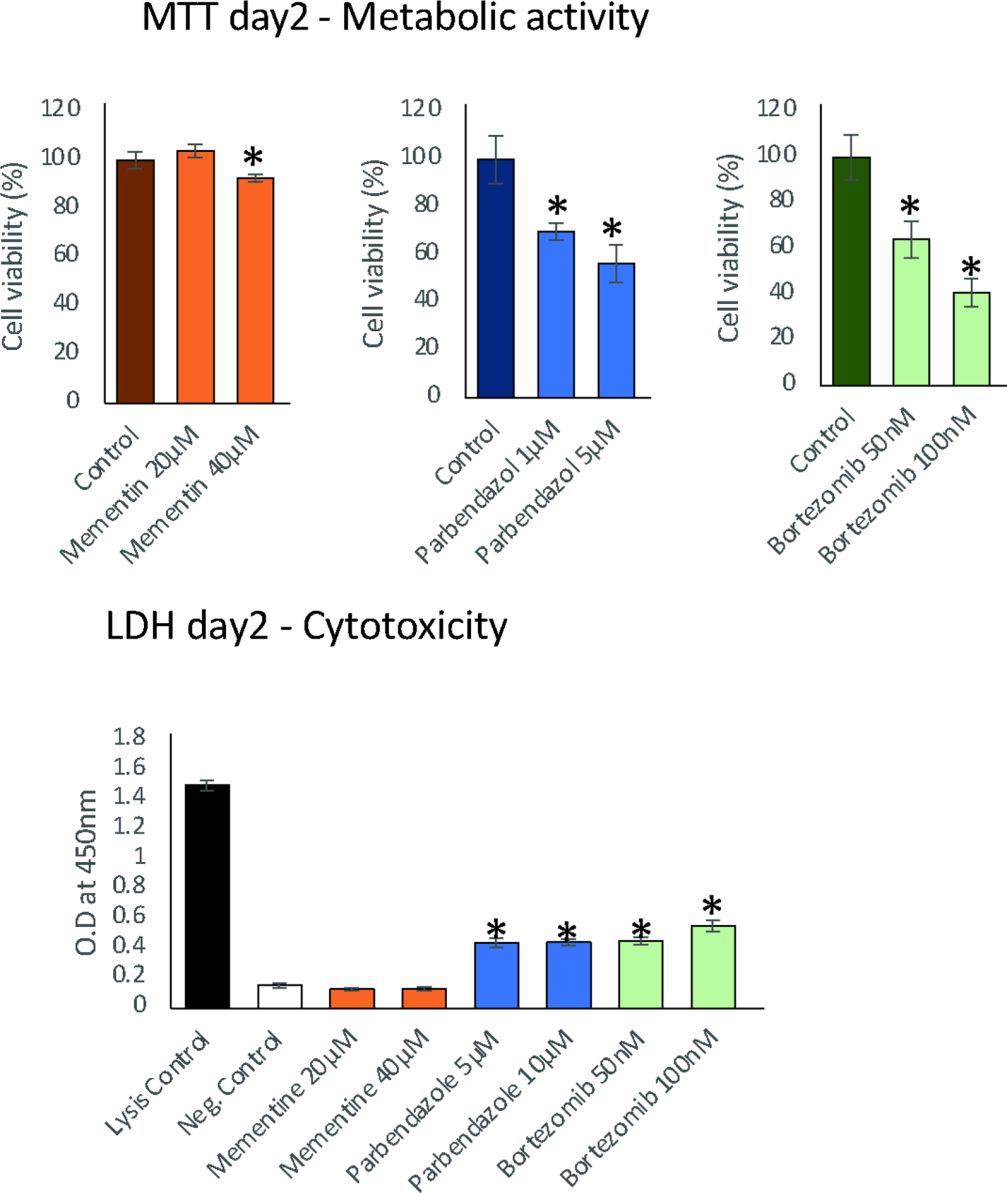
Bar plots showing the metabolic activity of drug-induced glial cells and cytotoxicity of memantine, parbendazole and bortezomib at different doses based on MTT assay and LDH release assay, after drug treatment on Day 2. A significant change in cell viability percentage representing metabolic activity compared to control or OD absorbance at 450 nm representing lysed cells compared to negative control is indicated by the * sign

**Fig. 6:**
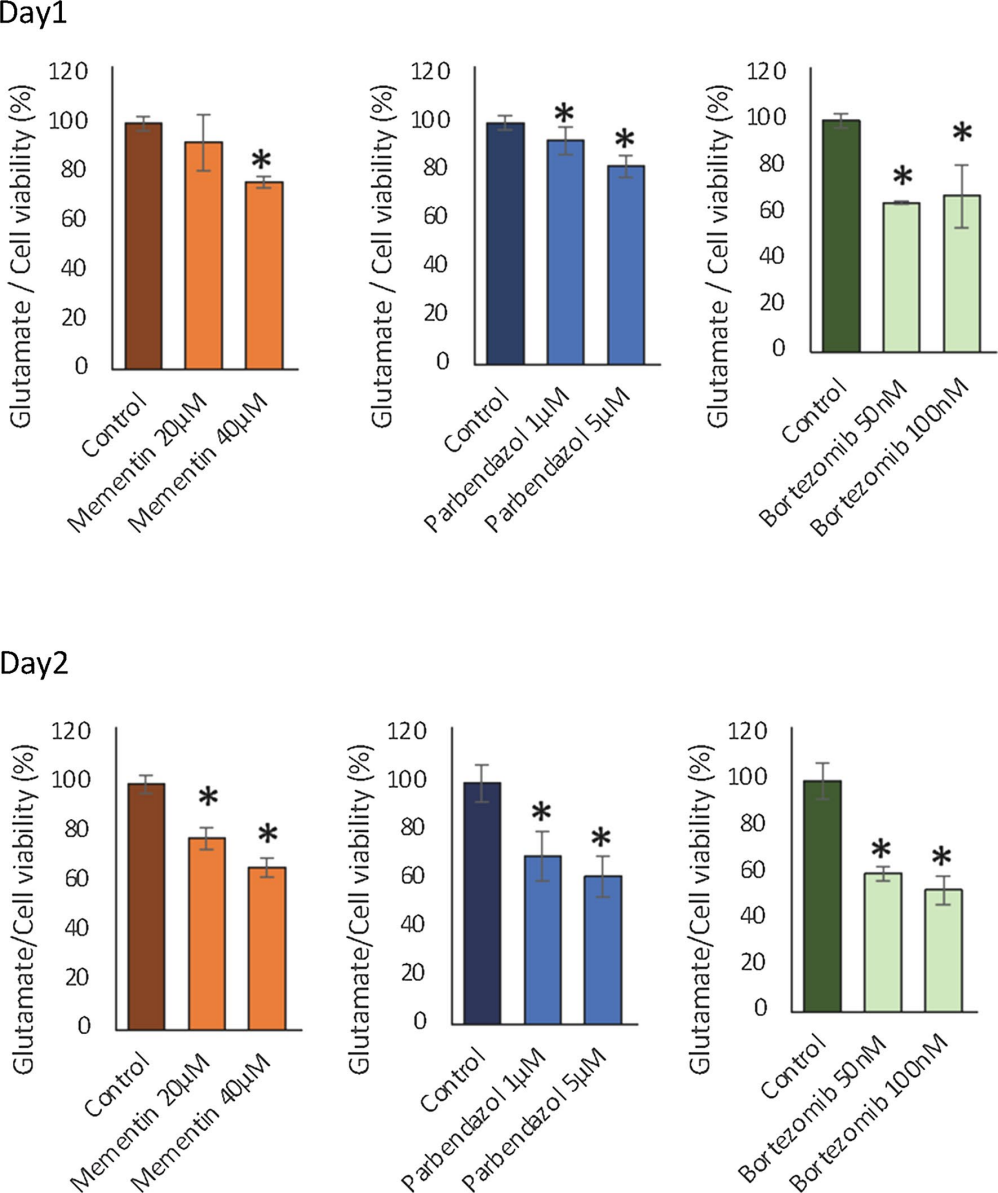
Bar plots showing the glutamate content in glial cells in comparison to cell viability for different doses of memantine, parbendazole and bortezomib, after drug treatment on Day 1 and Day 2. A significant change in the ratio compared to the control is indicated by the * sign

**Fig. 7:**
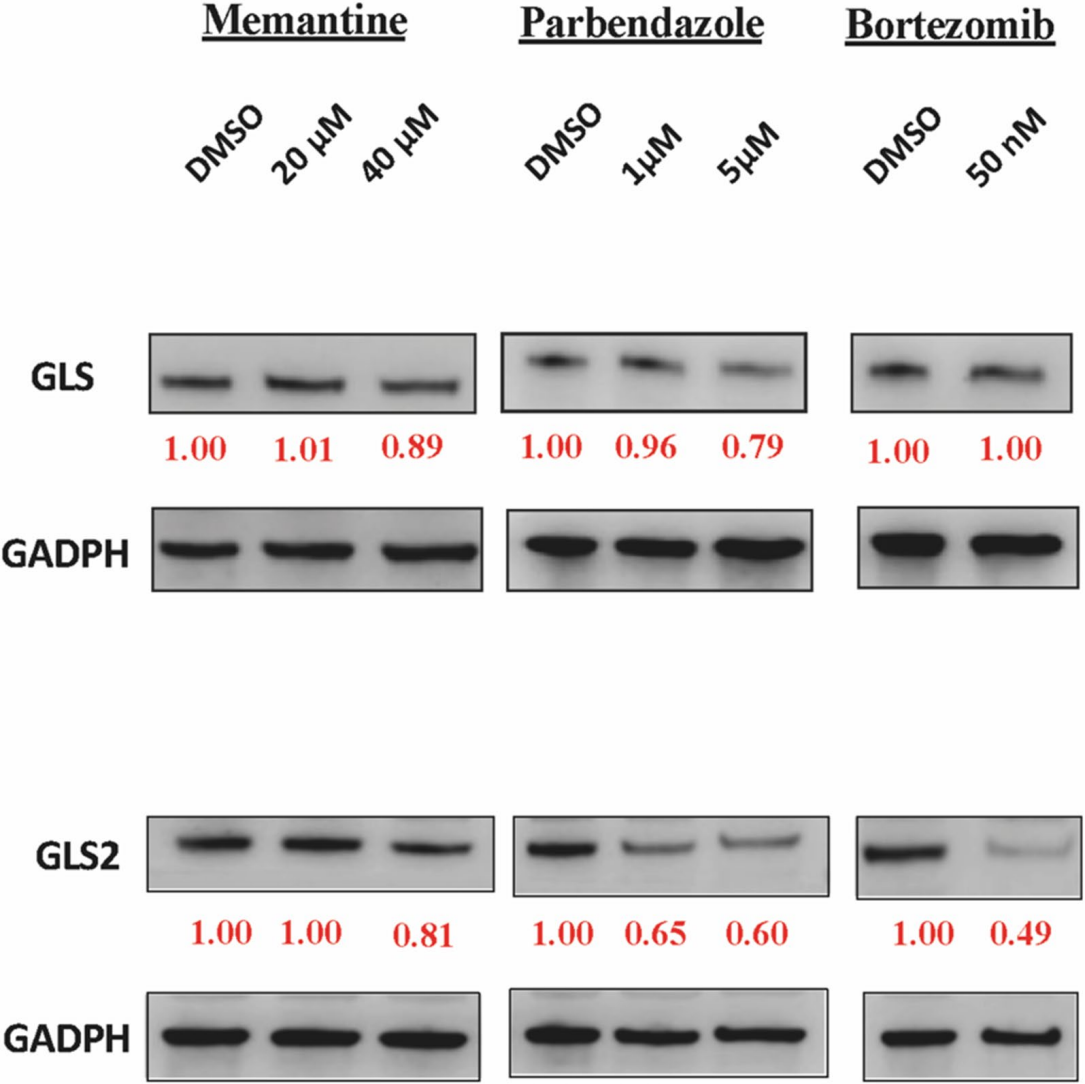
Western blot images showing the protein levels of GLS, GLS2, and GAPDH (negative control) after drug treatment on Day 2. Relative band intensities were quantified by Image J software and calculated in reference to controls of each drug–protein group, where DMSO band intensities are 1.00

Drug toxicity was considered by MTT and LDH to release assays, which are used to test metabolic activity and cytotoxicity, respectively. Both parbendazole and bortezomib were shown to be toxic for glial cells even at low concentrations, whereas memantine toxicity was insignificant when using 20 μm (Fig. 5). On the other hand, glutamate levels were highly decreased after the treatment of all three drugs, which are comparable when cell viability was considered (Fig. 6). This suggests that parbendazole and bortezomib may show their intended efficacy despite their known anti-apoptotic effects. Western blotting for glutaminase-1 (GLS) and glutaminase-2 (GLS2) was conducted to measure drug-induced levels for relative band intensities and find a direct or indirect interaction between drug and target as we depicted from drug-target network associations. Western blots showed that parbendazole and bortezomib significantly inhibited the protein levels of GLS and GLS2 (Fig. 7). Consequently, in vitro experiments proved computational estimations that suggested repurposable drugs reduce glutamate levels via suppression of glutaminase.

## Discussion

In this study, we identified *GLS* as a key gene for the treatment of AD based on GCN analysis, reporter metabolite analysis and essentiality analysis. We presented a profile-based drug repositioning method based on the correlation of gene knockdown or drug-induced transcription profiling of cell lines for AD treatment by suppressing the *GLS*-dependent synthesis of glutamate and glutamate signalling. By conducting an aggregated GSE analysis, investigating an interactome of DPIs/PPIs and performing in vitro experiments, we found functional and physical links between candidate drugs and *GLS*.

The conversion of glutamine to glutamate and other amino acids (i.e. glutaminolysis) is quite critical in ageing tissues. On the one hand, glutaminolysis occurs as a pH-balancing mechanism in the senescent cells and improves the survival [49]. On the other hand, the same mechanism may reduce the glutamate reuptake capacity of astrocytes, by inducing senescence. Further, this activates post-synaptic NMDARs, leading to potassium influx in neurons, and critically disrupts the ion balance [50]. Therefore, regulating glutaminases appears as an important checkpoint for elder health besides AD.

**Fig. 8:**
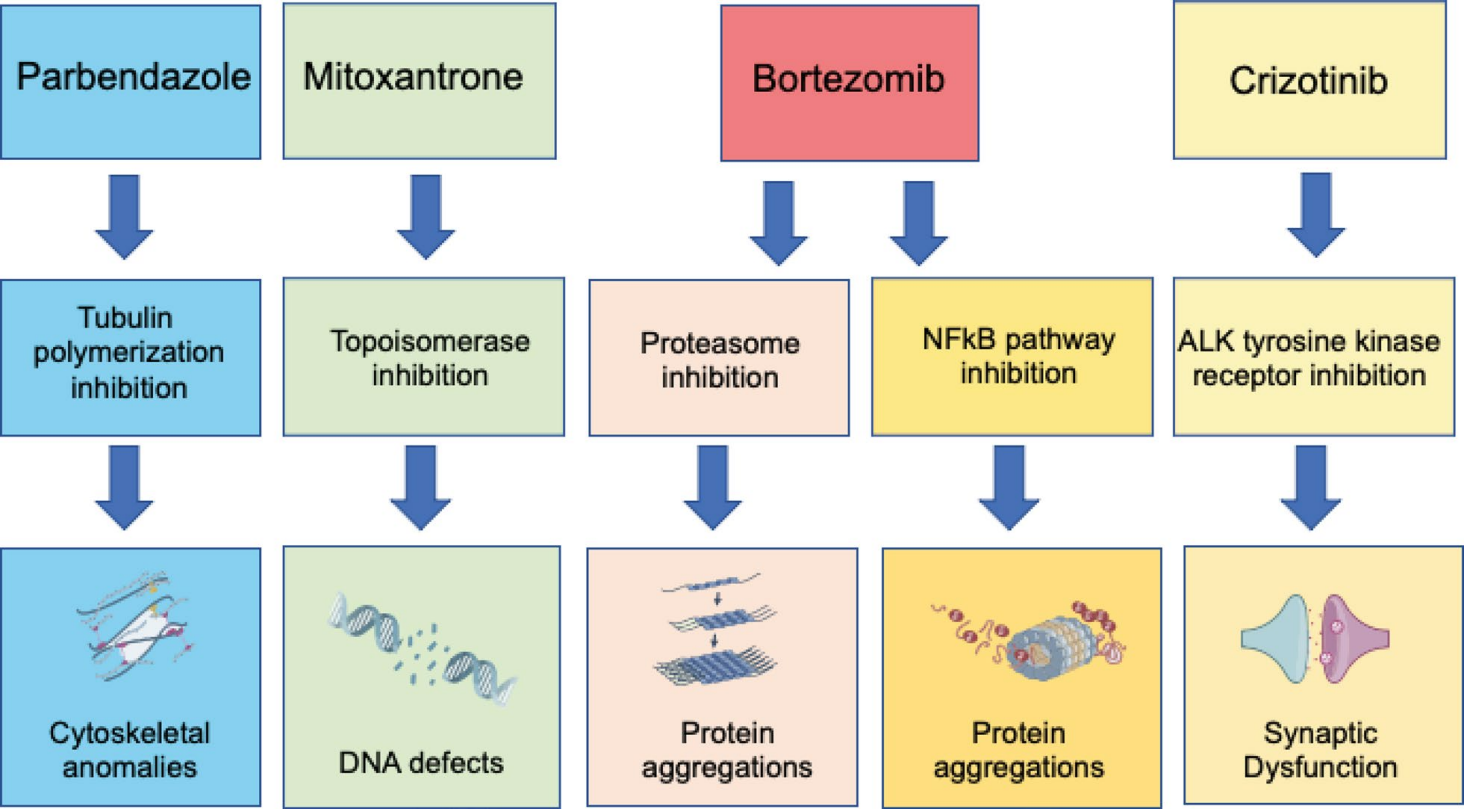
Visual representation of drug-induced MOA and affected neurodegenerative hallmarks

Eight candidate drugs were identified and further examined. Three of these compounds (BRD-K5433811, BRD-K03641750, SA-25547) are less studied. SA-25547 and BRD-K5433811 were predicted to be available to the brain based on their molecular structure, and the former had functional enrichments fitting glutaminase suppression. Still, they need to be tested experimentally further to elaborate their biological use. The other five drugs are well-studied anti-cancer drugs (bortezomib, crizotinib, mitoxantrone, withaferin-a) and one anthelmintic drug (parbendazole) whose mechanisms and adverse effects have been investigated. Aggregated gene set enrichments of these drugs showed great similarity to glutaminase suppression, implying their efficacy against glutaminase. A closer look at their targeting MOAs on interactome linked their strong effect on glutaminase balance via degradation and transcriptional activation. Here, we concluded that drug candidates carry the potential for targeting glutaminase isoforms via cell cycle regulatory elements and rebalance glutamate-induced disruption in ion homeostasis. Most importantly, drug MOAs are prominent components of neurodegenerative disease-defining hallmarks: tubulin polymerization inhibition to cytoskeletal abnormalities (Fig. 8) [51]. This underlines the centrality of glutamate synthesis in regard to AD causative mechanisms and their combined involvement in neurodegeneration. Finally, their ability as regulators of both glutaminase isoforms and glutamate levels was evidenced via in vitro experiments*.* The toxicity levels, however, enforce a second thought on the findings.

Cancer and neurodegeneration are often associated inversely, both sharing similarities on the opposite ends of biological events such as cell cycle [52, 53]. But accumulating pieces of evidence has caused a paradigm shift; many hallmarks of cancer can be common in the same direction as neurodegeneration, at the onset and during the progression of neurodegeneration, specifically for AD. Reduced AD risk in patients taking anti-cancer drug treatment and ongoing clinical trials on neurodegenerative disorders assist this shift in the cancer-neurodegeneration axis [54, 55]. Important cancer markers (e.g. p53, HIF1 and SIRT7) are demonstrated to induce glutaminase upregulation [56–58]. In addition, glutamate is often depicted as a major component of cancer metabolic reprogramming, considering the wide and crucial roles of glutamine and glutamate in energy production, amino acid biosynthesis and signal regulations; hence its isoforms have been targeted for the treatment of numerous cancer [59]. Although drug-target interactome could not show the full extent of transcription factors and signalling proteins affecting glutaminase, enrichment of many cancer signalling pathways (e.g., Ras, MAPK and JAK-STAT) underlines this strong interplay between glutaminase and these proteins and hereby associated with mitochondrial metabolism, immune response and cell proliferation.

Furthermore, there is evidence for the effect of these anti-cancer drugs on CNS. For instance, mitoxantrone has shown a positive effect on neurodegeneration in a longitudinal study of a multiple sclerosis cohort [60]. It has also been reported that withaferin-a activates the transcription of several cytoprotective enzymes, such as SOD1, CAT and GPX, and reduces the level of pro-inflammatory metabolites by inhibiting NF-kB activity that further attenuates TNF-a and COX-2 in rat brains [61].

Many other pieces of evidence are less clear for other drug candidates but still revolve around common cancer/AD hallmarks. Crizotinib inhibits MET tyrosine kinase receptor [62], whose native ligand, hepatocyte growth factor (HGF), induces extracellular signal-regulated kinase (ERK), and glutaminase indirectly. HGF/MET activation does not only affect glutaminases but also affects the various downstream proliferative signalling pathways. Moreover, HGF boosts glutamate receptor expression, synaptic plasticity and transmission in the hippocampal neurons [63]. Bortezomib is a proteasomal inhibitor and potentially an important modulator of autophagy [64], which is postulated to be vital for healthy ageing and treatment of AD [65]. Bortezomib also inhibits RELA (NF-kB subunit) and hence inactivates glutaminase.

Drug mechanisms are more complex and suggesting them as therapeutic targets directly is not suggestible for treating AD. To begin with, these anti-cancer drugs (i.e. mitoxantrone, withaferin-a, bortezomib and crizotinib) may not pass through BBB. Moreover, they are also anti-proliferative agents and they interact with receptors, enzymes and transcription factors not only initiating anti-inflammatory and neuroprotective events but also apoptotic events. For instance, topoisomerase inhibitors are quite selective but they also activate NFkB and alter anti-apoptotic or pro-inflammatory signalling pathways [66]. Proteasomal inhibitors may also impair mitochondrial function, increase inflammatory cytokines and elevate glutamate concentrations in contrast [67]. The anti-parasitic drug candidate parbendazole shows similar controversial properties, but BBB-permeability delivers a major advantage. Parbendazole degenerates microtubules of nematodes by targeting tubulin monomers as given in the interactome screening [68]. Conversely, immunocytochemistry screening of parbendazole and other anthelmintic drugs has shown increased myelin repair and oligodendroglial cell differentiation in rat and human cell cultures [69]. In addition to this proliferative effect, parbendazole may inhibit monoamine oxidase (MAO) and interfere with energy production [68]. MAO inhibitors inactivate monoamine neurotransmitters (e.g., dopamine, serotonin, melatonin) and they are used in psychiatric diseases, AD and PD [70, 71]. More interestingly, glutamate is often co-transmitted from all types of neurons together with primary neurotransmitters [72]; therefore, the use of MAO inhibitors or parbendazole may also regulate the imbalance in glutamate levels. Nevertheless, possible suppression of glutamatergic neuron microtubules may bring a risk of structural changes, and lead to a decrease in the transport of vesicles containing neurotransmitters to the synapse.

In this regard, bioengineering of these drugs is an almost inevitable necessity for their use in clinical practice. Replacement of unstable chemical substructures, the metabolic switching [73], the drug optimization [74], the development of prodrugs [75, 76], the drug conjugation [77] and the combination therapy [78, 79] are many proven strategies to resolve toxicity and BBB transport that can be suggested. Combinatorial therapeutic strategies are promising considering their links to evidenced AD hallmarks. As given in Table 3, we foresee the possibility of drug re-engineering based on computational assumptions. However, mitoxantrone may require extra consideration and withaferin-A may be difficult to re-engineer concerning its medicinal chemistry. This situation strengthens the importance of other candidate drugs due to their relative easiness.

**Table 3:** Table displaying the features of memantine and candidate drugs that are considerable in medicinal chemistry for optimization, produced by SwissADME

Molecule	PubChem Compound identifier	Formula	PAINS #alerts	Brenk #alerts	Leadlikeness #violations	Synthetic accessibility
Mitoxantrone	4212	C22H28N4O6	2	1	2	3.61
Crizotinib	11626560	C21H22Cl2FN5O	0	1	2	3.77
Withaferin-a	265237	C28H38O6	0	1	2	6.83
Bortezomib	387447	C19H25BN4O4	0	1	2	3.61
Parbendazole	26596	C13H17N3O2	0	0	1	2.17
BRD-54343811	118911863	C18H18N2O3	0	2	0	2.29
SA-25547	5337366	C20H15FN4O	2	1	0	3.36
Memantine	4054	C12H21N	0	0	1	3.7

The lower number of alerts for the PAINS filter indicates fewer substructures in a compound that respond or bind to proteins non-specifically, causing false positives in assays. The fewer alerts for the Brenk filter indicate fewer problematic fragments in a molecule that are putatively toxic, chemically reactive or metabolically unstable. The lower number of violations of leadlikeness indicates the easiness of converting a small lead-like molecule to another drug-like molecule. The lower synthetic accessibility, ranging between 1 and 10, indicates the estimated easiness of the molecule for synthetic optimization

## Conclusions

In a conclusion, high throughput gene expression profiling of distinct cell lines with and without exposure to drug candidates enabled the application of novel drug repositioning strategies and reveal the MOA of many drug candidates. We performed integrative analysis to reveal the druggability of several drugs against AD comparing their expression profiles to GLS knockdown. Then, we investigated their MOA relevant to glutaminase and determined their functional association with glutaminases. We finally validated the effect of parbendazole and bortezomib by performing in vitro experiments and found that these drugs could be used to target glutaminases and lower glutamate levels for the treatment of AD patients. Hence, the employment of a systems biology approach in the discovery of drug targets together with drug repositioning may be a promising strategy for the effective treatment of AD patients.

## Supplementary Information

**Additional file 1: Figure S1.** Boxplot for target gene (GLS) and other co-expressed genes’ essentiality scores from Chronos dataset on nervous system cell lines (n=114). Boxes represent the interquartile ranges where a median is the middle vertical line in a box. An essential score closer to -0.5 represents knockdown-dependent depletion and a score closer to -1 represents knockdown-dependent obliteration, whereas a zero score implies non-essentiality, and a positive score may indicate cell proliferation or may appear as a random annotation. **Figure S2.** The BOILED-Egg graphical output produced by SwissADME tool. The figure is demonstrating predicted pharmacokinetic features of compounds: passive gastrointestinal absorption (HIA), passive brain access (BBB) and active efflux from the central nervous system or to the gastrointestinal lumen by P-glycoproteins (PGP+: yes, PGP-: no) based on physicochemical descriptors (WLOGP and TPSA, for lipophilicity and apparent polarity). **Figure S3.** The distribution of the correlation coefficients for GLS-knockdown perturbed and drug-perturbed gene expression profiles for (left) the best four best top 1% drugs represented in at least two cell lines and memantine, (right) the best top 1% drugs represented six or all of seven cell lines. **Figure S4.** Bubble plots for aggregated pathway enrichments induced by (A) 100 best-correlated and (B) 100 randomly selected drugs, considering MSigDB hallmark pathways (n=50). Colour: red shows a larger normalized enrichment score (NES) / enrichment, and blue shows a larger negative NES / repression. Size: bigger “bubble” shows higher reliability, which is -log(FDR adjusted and weighted Fisher aggregated p-value) for enrichments. Shape: circle shows significant enrichments/repressions, triangle shows non-significant enrichment/repressions. **Figure S5.** Venn diagrams showing the overlaps for (left) entities and (right) interactions from CMap Repurposing App, Cheng’s paper (Cheng et al., 2018), custom interactome and HuRI database, respectively.

## Data Availability

The datasets used and/or analysed during the current study are available from the corresponding author upon reasonable request. Co-expression module gene network analysis results (modules_DLPFC.csv, modules_TCX.csv and modules_TCX.csv) and differential expression results (human_rosmap.csv, human_mayoTCX.csv, human_mayoCBE.csv) of that were used for target gene identification are available at [17]. Original CMap inputs (available at https://clue.io/data/CMap2020#LINCS2020, accessed on 16 November 2021): Level 5 shRNA-induced perturbations in GCTX format: level5_beta_trt_sh_n238351 × 12328.gctx. Level 5 compound-induced perturbations in GCTX: level5_beta_trt_cp_n720216 × 12328.gctx. Signature profile information including perturbation type, dose and time: siginfo_beta.txt. Cell line information including aliases, known targets and MOAs: cellinfo_beta.txt. Gene information including ENSEMBL IDs and source (n = 12328): geneinfo_beta.txt. Compound/drug information (n = 39321): compoundinfo_beta.txt. Metadata descriptions: LINCS2020_Release_Metadata_Field_Definitions.xlsx. Durg annotation-level information including relevant MOAs, target proteins, indications, diseases, disease areas and clinical trial phases for 6798 drugs (drug_activity.txt) was downloaded from CMap Repurposing App available at https://clue.io/repurposing-app. The inaccessible datasets used and/or analysed during the current study are available from the corresponding author upon reasonable request.

## References

[1] Ko Y (2020). Computational drug repositioning: current progress and challenges. Appl Sci.

[2] Jourdan JP, Bureau R, Rochais C, Dallemagne P (2020). Drug repositioning: a brief overview. J Pharm Pharmacol.

[3] Hascup KN, Findley CA, Britz J, Esperant-Hilaire N, Broderick SO, Delfino K (2021). Riluzole attenuates glutamatergic tone and cognitive decline in AβPP/PS1 mice. J Neurochem.

[4] Matthews DC, Mao X, Dowd K, Tsakanikas D, Jiang CS, Meuser C (2021). Riluzole, a glutamate modulator, slows cerebral glucose metabolism decline in patients with Alzheimer’s disease. Brain.

[5] Vossel K, Ranasinghe KG, Beagle AJ, La A, Ah Pook K, Castro M (2021). Effect of levetiracetam on cognition in patients with Alzheimer disease with and without epileptiform activity: a randomized clinical trial. JAMA Neurol.

[6] Mullane K, Williams M (2020). Alzheimer’s disease beyond amyloid: can the repetitive failures of amyloid-targeted therapeutics inform future approaches to dementia drug discovery?. Biochem Pharmacol.

[7] Clough E, Barrett T. The Gene Expression Omnibus database. In: Methods in Molecular Biology [Internet]. NIH Public Access; 2016 [cited 2022 Mar 28]. p. 93–110. Available from: /pmc/articles/PMC4944384/10.1007/978-1-4939-3578-9_5 PMC4944384 27008011

[8] Culhane AC, Schröder MS, Sultana R, Picard SC, Martinelli EN, Kelly C (2012). GeneSigDB: a manually curated database and resource for analysis of gene expression signatures. Nucleic Acids Res.

[9] Subramanian A, Narayan R, Corsello SM, Peck DD, Natoli TE, Lu X (2017). A next generation connectivity map: L1000 platform and the first 1,000,000 profiles. Cell.

[10] Cheng Z, Wen Y, Liang B, Chen S, Liu Y, Wang Z (2019). Gene expression profile-based drug screen identifies SAHA as a novel treatment for NAFLD. Mol Omi.

[11] Wu CH, Apweiler R, Bairoch A, Natale DA, Barker WC, Boeckmann B (2006). The universal protein resource (UniProt): an expanding universe of protein information. Nucleic Acids Res.

[12] Glicksberg BS, Li L, Chen R, Dudley J, Chen B (2019). Leveraging big data to transform drug discovery.

[13] Li X, Shong K, Kim W, Yuan M, Yang H, Sato Y (2022). Prediction of drug candidates for clear cell renal cell carcinoma using a systems biology-based drug repositioning approach. EBioMedicine.

[14] Yuan M, Shong K, Li X, Ashraf S, Shi M, Kim W (2022). A gene co-expression network-based drug repositioning approach identifies candidates for treatment of hepatocellular carcinoma. Cancers.

[15] Bonnet R, Mariault L, Peyron JF (2022). Identification of potentially anti-COVID-19 active drugs using the connectivity MAP. PLoS ONE.

[16] Nevado-Holgado AJ, Lovestone S (2017). Determining the molecular pathways underlying the protective effect of non-steroidal anti-inflammatory drugs for Alzheimer’s disease: a bioinformatics approach. Comput Struct Biotechnol J.

[17] Bayraktar A, Lam S, Altay O, Li X, Yuan M, Zhang C (2021). Revealing the molecular mechanisms of Alzheimer’s disease based on network analysis. Int J Mol Sci.

[18] Zhang C, Shi M, Kim W, Arif M, Klevstig M, Li X (2022). Discovery of therapeutic agents targeting PKLR for NAFLD using drug repositioning. EBioMedicine.

[19] Chen F, Guan Q, Nie ZY, Jin LJ (2013). Gene expression profile and functional analysis of Alzheimer’s disease. Am J Alzheimers Dis Other Demen.

[20] Rangaraju S, Dammer EB, Raza SA, Rathakrishnan P, Xiao H, Gao T (2018). Identification and therapeutic modulation of a pro-inflammatory subset of disease-associated-microglia in Alzheimer’s disease. Mol Neurodegener.

[21] Williams G, Gatt A, Clarke E, Corcoran J, Doherty P, Chambers D (2019). Drug repurposing for Alzheimer’s disease based on transcriptional profiling of human iPSC-derived cortical neurons. Transl Psychiatry.

[22] Lee H, Kang S, Kim W (2016). Drug repositioning for cancer therapy based on large-scale drug-induced transcriptional signatures. PLoS ONE.

[23] Dempster JM, Boyle I, Vazquez F, Root DE, Boehm JS, Hahn WC (2021). Chronos: a cell population dynamics model of CRISPR experiments that improves inference of gene fitness effects. Genome Biol.

[24] Enache OM, Lahr DL, Natoli TE, Litichevskiy L, Wadden D, Flynn C (2017). The GCTx format and cmap{Py, R, M} packages: resources for the optimized storage and integrated traversal of dense matrices of data and annotations. bioRxiv.

[25] Daina A, Michielin O, Zoete V (2017). SwissADME: a free web tool to evaluate pharmacokinetics, drug-likeness and medicinal chemistry friendliness of small molecules. Sci Rep.

[26] Baell JB, Holloway GA (2010). New substructure filters for removal of pan assay interference compounds (PAINS) from screening libraries and for their exclusion in bioassays. J Med Chem.

[27] Brenk R, Schipani A, James D, Krasowski A, Gilbert IH, Frearson J (2008). Lessons learnt from assembling screening libraries for drug discovery for neglected diseases. ChemMedChem.

[28] Teague SJ, Davis AM, Leeson PD, Oprea T (1999). The design of leadlike combinatorial libraries. Angew Chem Int Ed.

[29] Geistlinger L, Csaba G, Zimmer R (2016). Bioconductor’s enrichmentbrowser: seamless navigation through combined results of set- & network-based enrichment analysis. BMC Bioinform.

[30] Pagès H, Carlson M, Falcon S, Maintainer NL. Package ‘AnnotationDbi.’ Bioconductor Package Maintainer. 2017.

[31] Sergushichev AA (2016). An algorithm for fast preranked gene set enrichment analysis using cumulative statistic calculation. bioRxiv.

[32] Yoon S, Baik B, Park T, Nam D (2021). Powerful p-value combination methods to detect incomplete association. Sci Rep.

[33] Cheng F, Desai RJ, Handy DE, Wang R, Schneeweiss S, Barabási AL (2018). Network-based approach to prediction and population-based validation of in silico drug repurposing. Nat Commun.

[34] Luck K, Kim DK, Lambourne L, Spirohn K, Begg BE, Bian W (2020). A reference map of the human binary protein interactome. Nature.

[35] Csardi G, Nepusz T (2006). The igraph software package for complex network research. InterJournal.

[36] Gustavsen JA, Pai S, Isserlin R, Demchak B, Pico AR (2019). RCy3: network biology using cytoscape from within R. Research.

[37] Palmer AC, Kishony R (2014). Opposing effects of target overexpression reveal drug mechanisms. Nat Commun.

[38] Meyers RM, Bryan JG, McFarland JM, Weir BA, Sizemore AE, Xu H (2017). Computational correction of copy number effect improves specificity of CRISPR-Cas9 essentiality screens in cancer cells. Nat Genet.

[39] Jaladanki SK, Elmas A, Malave GS, HuangLin K (2021). Genetic dependency of Alzheimer’s disease-associated genes across cells and tissue types. Sci Rep.

[40] Márquez J, Matés JM, Campos-Sandoval JA (2016). Glutaminases. Adv Neurobiol.

[41] Walker MC, van der Donk WA (2016). The many roles of glutamate in metabolism. Physiol Behav.

[42] Haroon E, Miller AH, Sanacora G (2017). Inflammation, glutamate, and glia: a trio of trouble in mood disorders. Neuropsychopharmacology.

[43] Rumping L, Tessadori F, Pouwels PJW, Vringer E, Wijnen JP, Bhogal AA (2019). GLS hyperactivity causes glutamate excess, infantile cataract and profound developmental delay. Hum Mol Genet.

[44] Robinson DM, Keating GM (2006). Memantine: a review of its use in Alzheimer’s disease. Drugs.

[45] Kanehisa M, Goto S (2000). KEGG: kyoto encyclopedia of genes and genomes. Nucleic Acids Res.

[46] Zhou Y, Hou Y, Shen J, Huang Y, Martin W, Cheng F (2020). Network-based drug repurposing for novel coronavirus 2019-nCoV/SARS-CoV-2. Cell Discov.

[47] Fernández-Fernández MR, Gragera M, Ochoa-Ibarrola L, Quintana-Gallardo L, Valpuesta JM (2017). Hsp70—a master regulator in protein degradation. FEBS Lett.

[48] Hande KR (2008). Topoisomerase II inhibitors. Update Cancer Ther.

[49] Johmura Y, Yamanaka T, Omori S, Wang TW, Sugiura Y, Matsumoto M (2021). Senolysis by glutaminolysis inhibition ameliorates various age-associated disorders. Science.

[50] Sikora E, Bielak-Zmijewska A, Dudkowska M, Krzystyniak A, Mosieniak G, Wesierska M (2021). Cellular senescence in brain aging. Front Aging Neurosci.

[51] Wilson DMI, Cookson MR, Van Den BL, Zetterberg H, Holtzman DM (2023). Hallmarks of neurodegenerative diseases. Cell.

[52] Driver JA, Beiser A, Au R, Kreger BE, Splansky GL, Kurth T (2012). Inverse association between cancer and Alzheimer’s disease: results from the Framingham Heart Study. BMJ.

[53] Plun-Favreau H, Lewis PA, Hardy J, Martins LM, Wood NW (2010). Cancer and neurodegeneration: between the devil and the deep blue sea. PLoS Genet.

[54] Ancidoni A, Bacigalupo I, Remoli G, Lacorte E, Piscopo P, Sarti G (2021). Anticancer drugs repurposed for Alzheimer’s disease: a systematic review. Alzheimer’s Res Ther.

[55] Wrasidlo W, Crews LA, Tsigelny IF, Stocking E, Kouznetsova VL, Price D (2014). Neuroprotective effects of the anti-cancer drug sunitinib in models of HIV neurotoxicity suggests potential for the treatment of neurodegenerative disorders. Br J Pharmacol.

[56] Choudhury M, Yin X, Schaefbauer KJ, Kang JH, Roy B, Kottom TJ (2020). SIRT7-mediated modulation of glutaminase 1 regulates TGF-β-induced pulmonary fibrosis. FASEB J.

[57] Hu W, Zhang C, Wu R, Sun Y, Levine A, Feng Z (2010). Glutaminase 2, a novel p53 target gene regulating energy metabolism and antioxidant function. Proc Natl Acad Sci USA.

[58] Xiang L, Mou J, Shao B, Wei Y, Liang H, Takano N (2019). Glutaminase 1 expression in colorectal cancer cells is induced by hypoxia and required for tumor growth, invasion, and metastatic colonization. Cell Death Dis.

[59] Matés JM, Campos-sandoval JA, Santos-jiménez JDL, Segura JA, Alonso FJ, Márquez J (2019). Metabolic reprogramming of cancer by chemicals that target glutaminase metabolic reprogramming of cancer by chemicals that target glutaminase isoenzymes. Curr Med Chem.

[60] Chartier N, Epstein J, Soudant M, Dahan C, Michaud M, Pittion-Vouyovitch S (2018). Clinical follow-up of 411 patients with relapsing and progressive multiple sclerosis 10 years after discontinuing mitoxantrone treatment: a real-life cohort study. Eur J Neurol.

[61] Lee IC, Choi BY (2016). Withaferin-A—a natural anticancer agent with pleitropic mechanisms of action. Int J Mol Sci.

[62] Shaw AT, Yasothan U, Kirkpatrick P (2011). Crizotinib. Nat Rev Drug Discov.

[63] Sharma SK (2010). Hepatocyte growth factor in synaptic plasticity and Alzheimer ’ s disease. Sci World J.

[64] Eshraghi M, Ahmadi M, Afshar S, Lorzadeh S, Adlimoghaddam A, Rezvani Jalal N (2022). Enhancing autophagy in Alzheimer’s disease through drug repositioning. Pharmacol Ther.

[65] Aunan JR, Cho WC, Søreide K (2017). The biology of aging and cancer: a brief overview of shared and divergent molecular hallmarks. Aging Dis.

[66] Imre G. Anti-apoptotic and Pro-inflammatory Signaling in Cancer Cells : Status and Modulation by Chemotherapeutic Drugs. 2007. https://kops.uni-konstanz.de/server/api/core/bitstreams/81bf6357-fe5e-4343-a8da-c93a8224f505/content.

[67] Yamamoto S, Egashira N (2021). Pathological mechanisms of bortezomib-induced peripheral neuropathy. Int J Mol Sci.

[68] Al-Hadiya BMH (2010). Parbendazole.

[69] Manousi A, Göttle P, Reiche L, Cui QL, Healy LM, Akkermann R (2021). Identification of novel myelin repair drugs by modulation of oligodendroglial differentiation competence. EBioMedicine.

[70] Dezsi L, Vecsei L (2017). Monoamine oxidase B inhibitors in Parkinson’s disease. CNS Neurol Disord Drug Targets.

[71] Finberg JPM, Rabey JM (2016). Inhibitors of MAO-A and MAO-B in psychiatry and neurology. Front Pharmacol.

[72] Trudeau LE, El Mestikawy S (2018). Glutamate cotransmission in cholinergic, GABAergic and monoamine systems: contrasts and commonalities. Front Neural Circuits.

[73] Teleanu RI, Preda MD, Niculescu AG, Vladâcenco O, Radu CI, Grumezescu AM (2022). Current strategies to enhance delivery of drugs across the blood-brain barrier. Pharmaceutics.

[74] Yukawa E (1996). Optimisation of antiepileptic drug therapy. Clin Pharmacokinet.

[75] Meng F, Evans JW, Bhupathi D, Banica M, Lan L, Lorente G (2012). Molecular and cellular pharmacology of the hypoxia-activated prodrug TH-302. Mol Cancer Ther.

[76] Lindquist KE, Cran JD, Kordic K, Chua PC, Winters GC, Tan JS (2013). Selective radiosensitization of hypoxic cells using BCCA621C: a novel hypoxia activated prodrug targeting DNA-dependent protein kinase. Tumor Microenviron Ther.

[77] Pardridge WM (2012). Drug transport across the blood-brain barrier. J Cereb Blood Flow Metab.

[78] Luszczki JJ, Mazurkiewicz LP, Wroblewska-Luczka P, Wlaz A, Ossowska G, Szpringer M (2018). Combination of phenobarbital with phenytoin and pregabalin produces synergy in the mouse tonic-clonic seizure model: an isobolographic analysis. Epilepsy Res.

[79] Juraszek B, Nałąecz KA (2020). SLC22A5 (OCTN2) carnitine transporter-indispensable for cell metabolism, a jekyll and hyde of human cancer. Molecules.

