# Supplementary material for: Drug repositioning targeting glutaminase reveals drug candidates for the treatment of Alzheimer’s disease patients
Source: J Transl Med. 2023 May 20;21:332. doi: 10.1186/s12967-023-04192-6 (PMC10199278; doi:10.1186/s12967-023-04192-6)
Supplement: Supplementary file 1 — Additional file 1: Figure S1. Boxplot for target gene (GLS) and other co-expressed genes’ essentiality scores from Chronos dataset on nervous system cell lines (n=114). Boxes represent the interquartile ranges where a median is the middle vertical line in a box. An essential score closer to -0.5 represents knockdown-dependent depletion and a score closer to -1 represents knockdown-dependent obliteration, whereas a zero score implies non-essentiality, and a positive score may indicate cell proliferation or may appear as a random annotation. Figure S2. The BOILED-Egg graphical output produced by SwissADME tool. The figure is demonstrating predicted pharmacokinetic features of compounds: passive gastrointestinal absorption (HIA), passive brain access (BBB) and active efflux from the central nervous system or to the gastrointestinal lumen by P-glycoproteins (PGP+: yes, PGP-: no) based on physicochemical descriptors (WLOGP and TPSA, for lipophilicity and apparent polarity). Figure S3. The distribution of the correlation coefficients for GLS-knockdown perturbed and drug-perturbed gene expression profiles for (left) the best four best top 1% drugs represented in at least two cell lines and memantine, (right) the best top 1% drugs represented six or all of seven cell lines. Figure S4. Bubble plots for aggregated pathway enrichments induced by (A) 100 best-correlated and (B) 100 randomly selected drugs, considering MSigDB hallmark pathways (n=50). Colour: red shows a larger normalized enrichment score (NES) / enrichment, and blue shows a larger negative NES / repression. Size: bigger “bubble” shows higher reliability, which is -log(FDR adjusted and weighted Fisher aggregated p-value) for enrichments. Shape: circle shows significant enrichments/repressions, triangle shows non-significant enrichment/repressions. Figure S5. Venn diagrams showing the overlaps for (left) entities and (right) interactions from CMap Repurposing App, Cheng’s paper (Cheng et al., 2018), custom intera [file 12967_2023_4192_MOESM1_ESM.pdf]

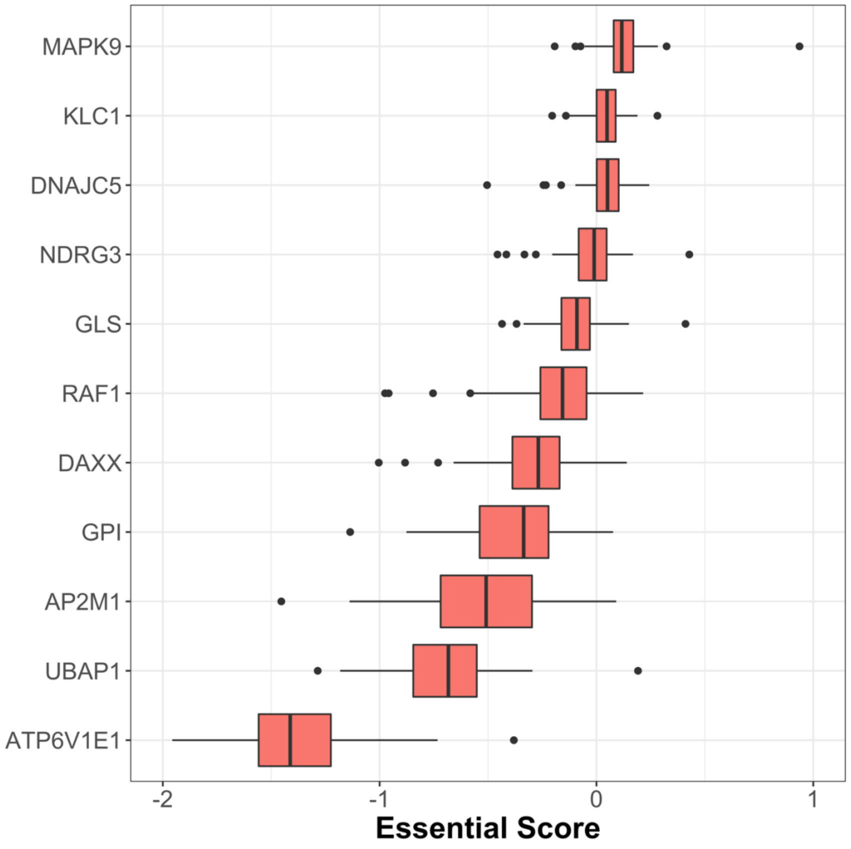

Figure 1: Boxplot for target gene (GLS) and other co-expressed genes' essentiality scores from Chronos dataset on nervous system cell lines (n=114). Boxes represent the interquartile ranges where a median is the middle vertical line in a box. An essential score closer to -0.5 represents knockdown-dependent depletion and a score closer to -1 represents knockdown-dependent obliteration, whereas a zero score implies non-essentiality, and a positive score may indicate cell proliferation or may appear as a random annotation.

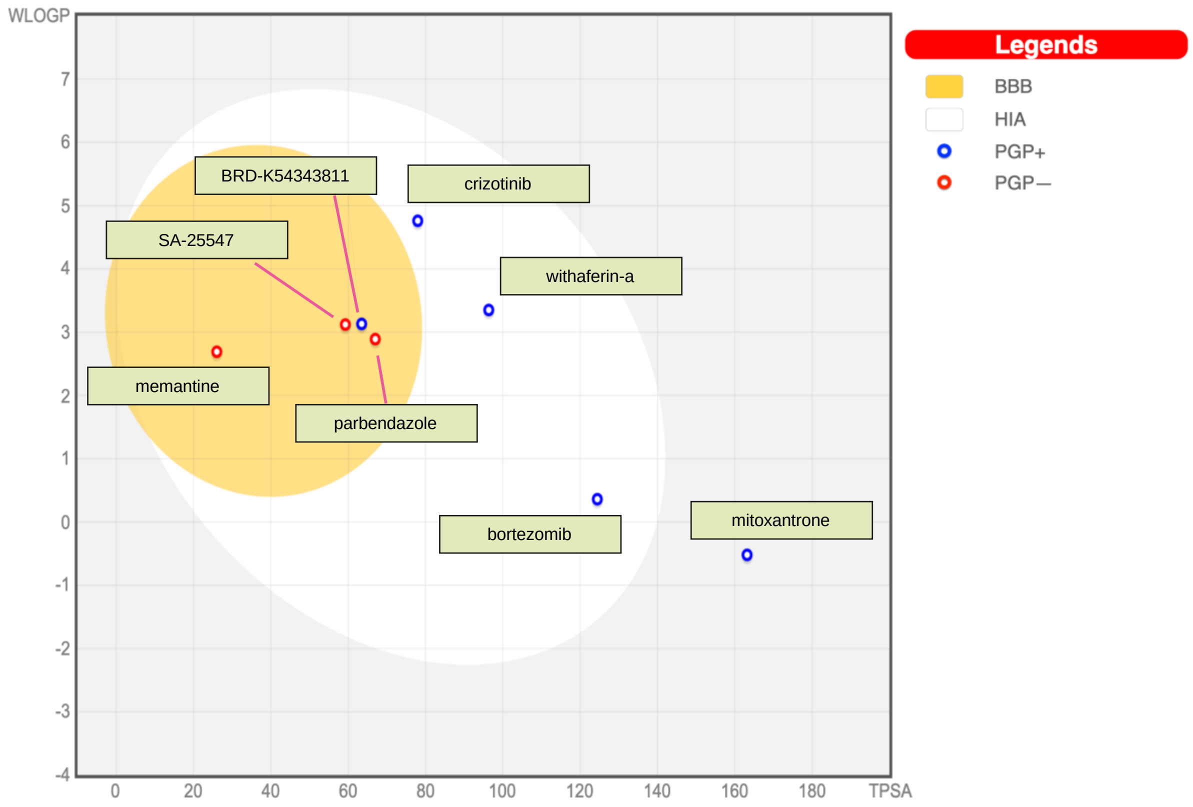

Figure 2: the BOILED-Egg graphical output produced by SwissADME tool. The figure is demonstrating predicted pharmacokinetic features of compounds: passive gastrointestinal absorption (HIA), passive brain access (BBB) and active efflux from the central nervous system or to the gastrointestinal lumen by P-glycoproteins (PGP+: yes, PGP-: no) based on physicochemical descriptors (WLOGP and TPSA, for lipophilicity and apparent polarity)

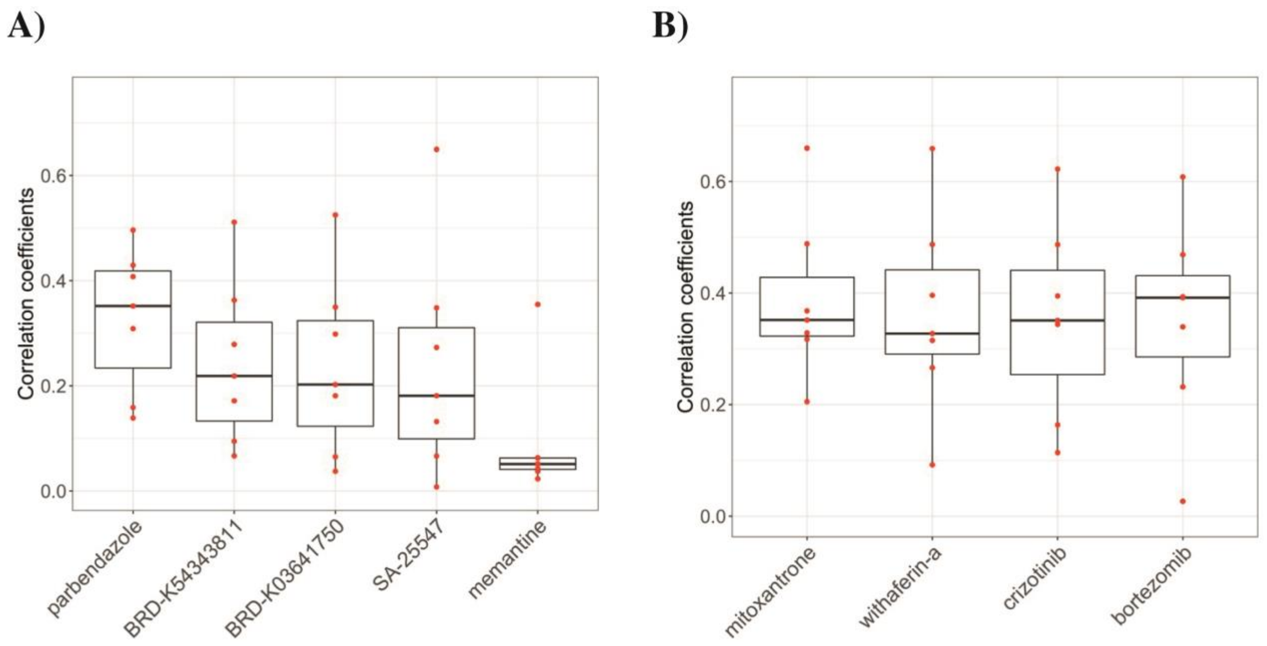

Figure 3: The distribution of the correlation coefficients for GLS-knockdown perturbed and drug-perturbed gene expression profiles for (left) the best four best top 1% drugs represented in at least two cell lines and memantine, (right) the best top 1% drugs represented six or all of seven cell lines.

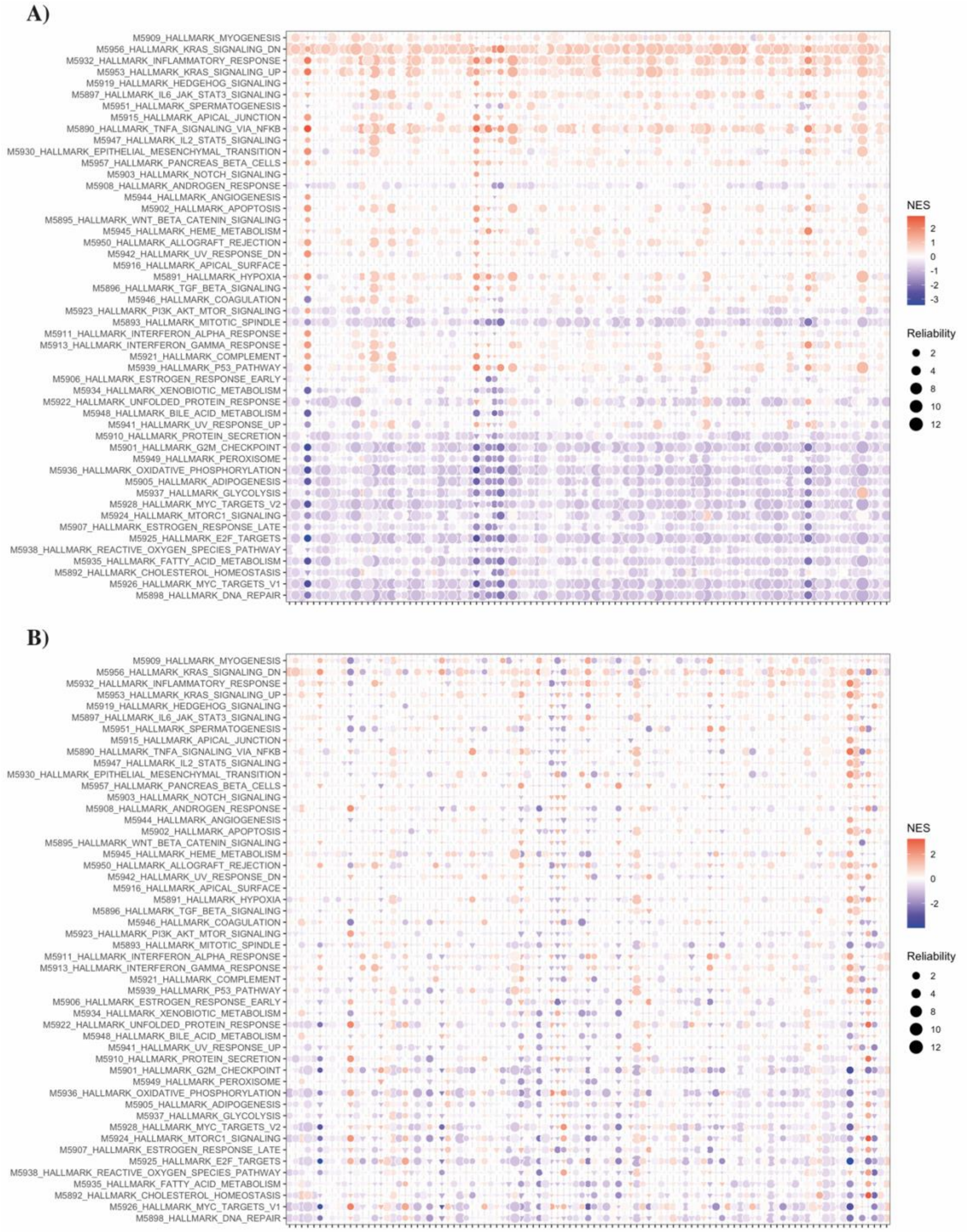

Figure 4: Bubble plots for aggregated pathway enrichments induced by (A) 100 best-correlated and (B) 100 randomly selected drugs, considering MSigDB hallmark pathways (n=50). Colour: red shows a larger normalized enrichment score (NES) / enrichment, and blue shows a larger negative NES / repression. Size: bigger “bubble” shows higher reliability, which is -log(FDR adjusted and weighted Fisher aggregated p-value) for enrichments. Shape: circle shows significant enrichments/repressions, triangle shows non-significant enrichment/repressions.

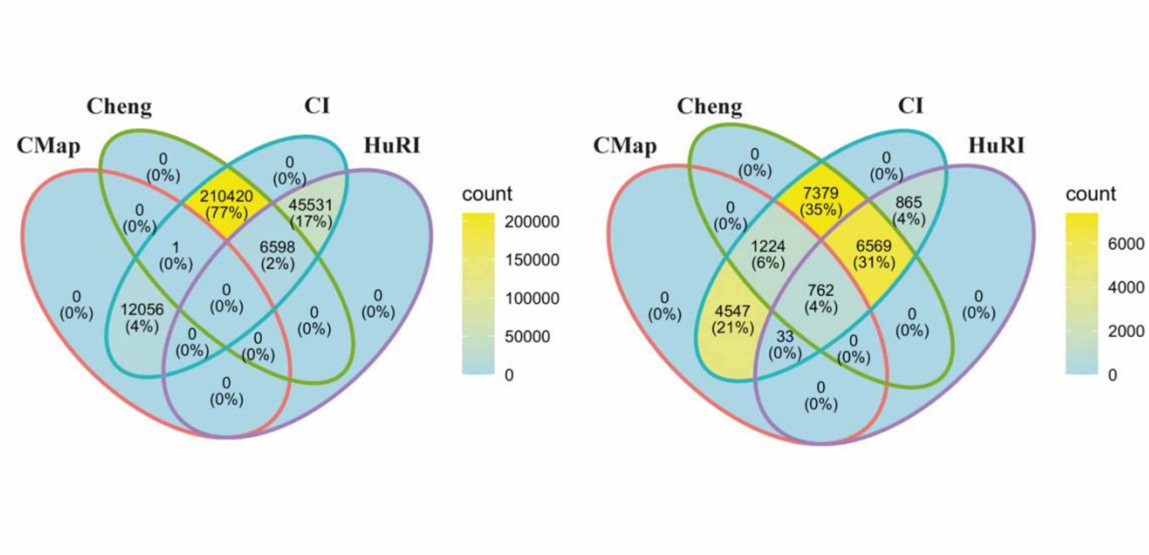

Figure 5: Venn diagrams showing the overlaps for (left) entities and (right) interactions from CMap Repurposing App, Cheng's paper (Cheng et al., 2018), custom interactome and HuRI database, respectively.
